# Strong activation of p53 by actinomycin D and nutlin-3a overcomes the resistance of cancer cells to the pro-apoptotic activity of the FAS ligand

**DOI:** 10.1007/s10495-024-02000-0

**Published:** 2024-07-28

**Authors:** Barbara Łasut-Szyszka, Agnieszka Gdowicz-Kłosok, Małgorzata Krześniak, Magdalena Głowala-Kosińska, Agnieszka Będzińska, Marek Rusin

**Affiliations:** 1 Center for Translational Research and Molecular Biology of Cancer, Gliwice Branch, Maria Skłodowska-Curie National Research Institute of Oncology, ul. Wybrzeże Armii Krajowej 15, Gliwice, 44-101 Poland; 2 Department of Bone Marrow Transplantation and Onco-Hematology, Gliwice Branch, Maria Skłodowska-Curie National Research Institute of Oncology, Gliwice, 44-101 Poland

**Keywords:** p53, FASLG, Apoptosis, Death receptor, MDM2, Cancer immunotherapy

## Abstract

The FAS ligand (FASLG) is expressed on lymphocytes, which employ it to activate death receptors on target cells. Cancer cells are generally resistant to apoptosis triggered by FASLG. In this work, we found a way to circumvent this resistance by treatment with actinomycin D (ActD) and nutlin-3a (Nut3a). We selected this drug combination based on our transcriptomic data showing strong activation of proapoptotic genes, including those for receptor-mediated apoptosis, in cells exposed to actinomycin D and nutlin-3a. To test our hypothesis, we pre-exposed cancer cell lines to this drug combination for 45 h and then treated them with recombinant FASLG. This almost instantaneously killed most cells. Actinomycin D and nutlin-3a strongly cooperated in the sensitization because the effect of the drugs acting solo was not as spectacular as the drug combination, which together with FASLG killed more than 99% of cells. Based on the caspase activation pattern (caspase-8, caspase-9, caspase-10), we conclude that both extrinsic and intrinsic pro-apoptotic pathways were engaged. In engineered p53-deficient cells, this pro-apoptotic effect was completely abrogated. Therefore, the combination of ActD + Nut3a activates p53 in an extraordinary way, which overcomes the resistance of cancer cells to apoptosis triggered by FASLG. Interestingly, other combinations of drugs, e.g., etoposide + nutlin-3a, actinomycin D + RG7112, and actinomycin D + idasanutlin had a similar effect. Moreover, normal human fibroblasts are less sensitive to death induced by ActD + Nut3a + FASLG. Our findings create the opportunity to revive the abandoned attempts of cancer immunotherapy employing the recombinant FAS ligand.

**Graphical Abstract:**

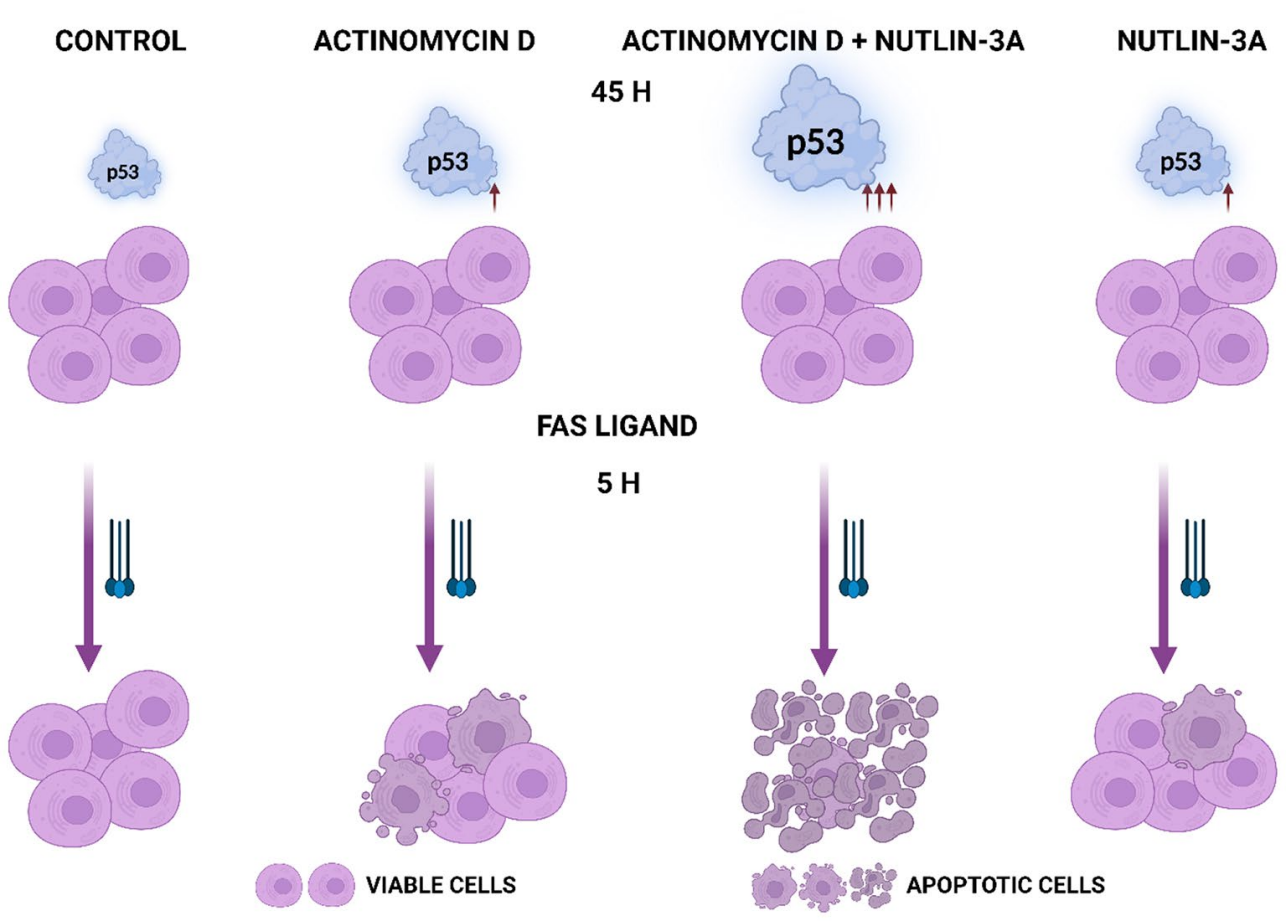

**Supplementary Information:**

The online version contains supplementary material available at 10.1007/s10495-024-02000-0.

## Introduction

One of the best-studied functions of p53 is the induction of apoptosis. P53 performs this task by directly activating the expression of many pro-apoptotic genes that encode proteins located at various points of programmed-cell-death signaling cascades starting either from death receptors (extrinsic pathway) or from the detectors of internal cellular stress like DNA damage (intrinsic or mitochondrial pathway) (reviewed by Aubrey et al. [1]).

The apoptosis triggered by death receptors plays an important role in the functioning of the immune system, for example, when the cytotoxic lymphocytes or natural killer cells destroy their targets (e.g., incipient cancer cells) by stimulating death receptors on their surface. The death receptors are characterized by an extracellular domain, which binds to their cognate ligands, and by an intracellular “death domain”, recruiting various proteins that mediate cell death. One of these proteins is called FADD, which binds to the death domain of receptors and the death effector domain of inactive caspase-8. This aggregate of death receptor, FADD, and caspase-8 is called DISC (death-inducing signaling complex). Through a complicated process, the inactive caspase-8 in DISC is cleaved into smaller fragments and forms active caspase-8 consisting of two smaller and two larger subunits. In some cell types (e.g., immune cells), the activation of death receptors leads to the formation of so many active caspase-8 molecules that they are able to directly activate caspase-3, which is the major executioner of apoptosis. In other cell types (e.g., hepatocytes), the active caspase-8, in order to trigger apoptosis, must first engage the intrinsic mitochondrial pathway with all its consequences: increased permeability of the outer mitochondrial membrane, release of cytochrome c, formation of apoptosome, activation of caspase-9, and finally, the activation of caspase-3 (reviewed by Fouque et al. [2], Green and Llambi [3]).

One of the death receptor genes activated by p53 is called *FAS* [4]. The activation of *FAS* expression is a part of the pro-apoptotic, p53-dependent transcriptional program. A previous study demonstrated that anticancer drugs could sensitize cancer cells to FAS-mediated cytotoxicity [5]. Anticancer drugs can sensitize cancer cells to the FAS agonist by activating p53, which in turn promotes the expression of the FAS receptor and probably other pro-apoptotic proteins.

In our previous work, we demonstrated that two p53 activators, actinomycin D and nutlin-3a, synergize in the activation of p53 and consequently in inducing the expression of some p53-regulated genes [6]. The mechanism of this synergy is not known. We hypothesize that nutlin-3a (Nut3a), which antagonizes the negative regulator of p53 – the MDM2 protein, makes p53 a better target for activating kinases triggered by actinomycin D (ActD). Whatever the mechanism, in A549 lung cancer cells, the co-treatment with ActD + Nut3a strongly stimulates the expression of many p53-regulated pro-apoptotic genes, including the FAS receptor gene and caspase-10 gene [7]. However, despite very strong upregulation of pro-apoptotic genes, the apoptotic cells in the cell population exposed to ActD + Nut3a are infrequent [8]; apparently, a crucial trigger for apoptosis is missing. We started the current work by testing the hypothesis that co-treatment with ActD + Nut3a sensitizes the cancer cells, in a p53-dependent manner, to the apoptosis triggered by the FAS ligand (FASLG).

### Materials and methods

#### Cell culture and treatment

We used early passage cell lines obtained from ATCC. A549 cells (RRID: CVCL_0023, lung adenocarcinoma, American Type Culture Collection [ATCC]), and U-2 OS cells (RRID: CVCL_0042, osteosarcoma, ATCC) were cultured in low glucose DMEM supplemented with 10% fetal bovine serum (FBS; Invitrogen, Carlsbad, CA, USA). NCI-H460 (RRID: CVCL_0459, lung cancer, ATCC) were cultured in RPMI-1640 supplemented with 2 mM L-glutamine, 4.5 g/L glucose, 1 mM sodium pyruvate, and 10% heat-inactivated FBS. AGS cells (RRID: CVCL_0139, gastric adenocarcinoma, ATCC) were cultured in McCoy′s 5 A supplemented with 10% fetal bovine serum. Jurkat cells were cultured in RPMI-1640 supplemented with 2 mM L-glutamine, 2 g/L glucose, 1 mM sodium pyruvate and 10% FBS. Normal human fibroblasts (GM07492, Coriel Cell Repositories, Camden, NJ) of early passage were cultured in low glucose DMEM supplemented with 15% FBS. All cells were cultured at 37 °C/5% CO2 and all media were supplemented with a 1% penicillin/streptomycin solution.

The stock solutions of chemicals were prepared in DMSO, being actinomycin D (10 µM; Sigma-Aldrich, St. Louis, MI, USA), nutlin-3a (10 mM; Selleck Chemicals LLC, Houston, TX, USA), idasanutlin (10mM), RG7112 (10 mM), Z-VAD-FMK (2 mM, cat. no. HY-16658B, pan caspase inhibitor) and Z-IETD-FMK (2 mM, cat no. HY-101297, caspase-8 inhibitor; MedChemExpress LLC, Monmouth Junction, NJ, USA). The stock solution of etoposide (20 mg/ml, 34 mM, concentrate for solution for infusion) was obtained from Accord Healthcare Poland, Warsaw. The stock solutions were diluted in culture medium at the following concentrations: 5 nM actinomycin D, 5 µM nutlin-3a, 5 µM RG7112, 5 µM Idasanutlin, 100 µM Z-VAD-FMK, 100 µM Z-IETD-FMK and 5 or 25 µM etoposide as indicated in the Results section. Control cells were mock-treated with a medium containing DMSO. The human FAS ligand (His Tag, active trimer, cat. no. FAL-H5241) was purchased from Acro Biosystems (Newark, DE, USA). To prepare the stock solution, the lyophilized product was reconstituted in deionized water to a solution of 1000–200 µg/ml (as suggested by the manufacturer depending on the lot).

*Live-cell imaging*.

The cells were seeded on chambered coverglass. The next day, they were exposed to ActD + Nut3a or DMSO (Control - mock-treated). After 48 h of incubation, the media (with ActD + Nut3a or control) were removed and the cells were exposed to FASLG (100 ng/ml) or control medium. Subsequently, the dish with cells was transferred to a heating insert with a CO_2_ cover on the stage of an inverted microscope (ZEISS Elyra 7 with Lattice SIM²). Images were taken from the same field using a 20x objective. The images were acquired using standard software (ZEN black edition, Carl Zeiss), and the images were processed using ZEN 3.1 (ZEN Lite, Carl Zeiss).

#### Cell staining on culture plates

An equal number of cells were seeded on 12-well culture plates at a calculated 10% confluence. After overnight incubation, the cells were treated with indicated drugs or mock-treated for approximately 45 h. Subsequently, fresh culture medium (with or without FASLG) was added and the cells were incubated for 5 h. After this, the medium was changed again, and the cells were allowed to recover for 24–70 h as indicated in the Results. The medium was removed, and the cells were fixed by incubation with − 20^o^C methanol for 10 min. Dried cells were stained with 0.01% crystal violet for 2 min. Finally, the cells were washed with distilled water, dried, and the plates were scanned.

#### Colorimetric cell viability assay

A549 cells were seeded (5 000/well) on 96-well plates. After drug treatment (ActD, ActD + Nut3a, Nut3a) for 45 h, the cells were either mock-treated or incubated with FASLG (at indicated concentrations for 5 h). After this, the medium was replaced for 24 h of recovery time. Next, cell viability was determined using a CellTiter 96^®^ AQueous One Solution Cell Proliferation Assay (MTS) (Promega) kit according to the manufacturer’s protocol. The absorbance was read at 490 nm using a microplate reader. Data were normalized to the control (no drug or FASLG treatment). Four independent biological replicates were performed.

#### Western blotting

Whole-cell lysates were prepared using an IP buffer, supplemented with protease and phosphatase inhibitors as described previously [6]. Aliquots of lysates (15–40 µg) were separated by SDS-PAGE on 8% or 13% gels and then electro-transferred onto PVDF membranes. Before incubation with a primary antibody, the membranes were incubated for 1 h at room temperature in blocking solution (5% skim milk in PBS with 0.1% Tween-20). Anti-cleaved caspase-3 (Asp175) (5A1E) rabbit mAb, anti-caspase-8 (1C12) mouse mAb, anti-caspase-9 polyclonal rabbit Ab, anti-FAS rabbit mAb (C18C12), and anti-Apaf-1 (D5C3) rabbit mAb were obtained from Cell Signaling Technology (Danvers, MA, USA). The anti-caspase-10 rabbit polyclonal antibody and anti-caspase-6 mouse monoclonal antibody were obtained from Proteintech (Rosemont, IL, USA). Anti-p53 (DO-1) and loading control anti-HSC70 (B-6) antibodies were obtained from Santa Cruz Biotechnology (Dallas, TX, USA). All incubations with primary antibodies were performed overnight at 4^o^C in blocking solution. HRP-conjugated secondary antibodies (anti-mouse, anti-rabbit) were detected by chemiluminescence (SuperSignalWest Pico or SuperSignal West Femto Chemiluminescent Substrate, Thermo Fisher Scientific, Waltham, MA, USA).

#### Flow cytometry

The apoptotic cells were analyzed using a PE Annexin V Apoptosis Detection Kit I (BD Biosciences, San Jose, CA, USA) according to the manufacturer’s protocol using a BD FACSCanto™ cytometer (Becton Dickinson, San Jose, CA, USA). The data were analyzed using BD FACSDiva™ software. The expression of FASLG (CD95L) on the cell surface was determined using BD Pharmingen™ APC Mouse Anti-Human CD95 (Becton, Dickinson and Company, NJ, USA) and APC Mouse IgG1 kappa Isotype Control (P3.6.2.8.1) (eBioscience™, San Diego, USA).

#### Statistical analysis

The graphs and statistical significance of the differences analyzed in cell viability or death (apoptosis/necrosis) were calculated using GraphPad Prism version 10.0.0 for Windows, GraphPad Software, Boston, Massachusetts USA, www.graphpad.com. The employed tests and the level of significance are presented in the Results.

## Results

**Fig. 1:**
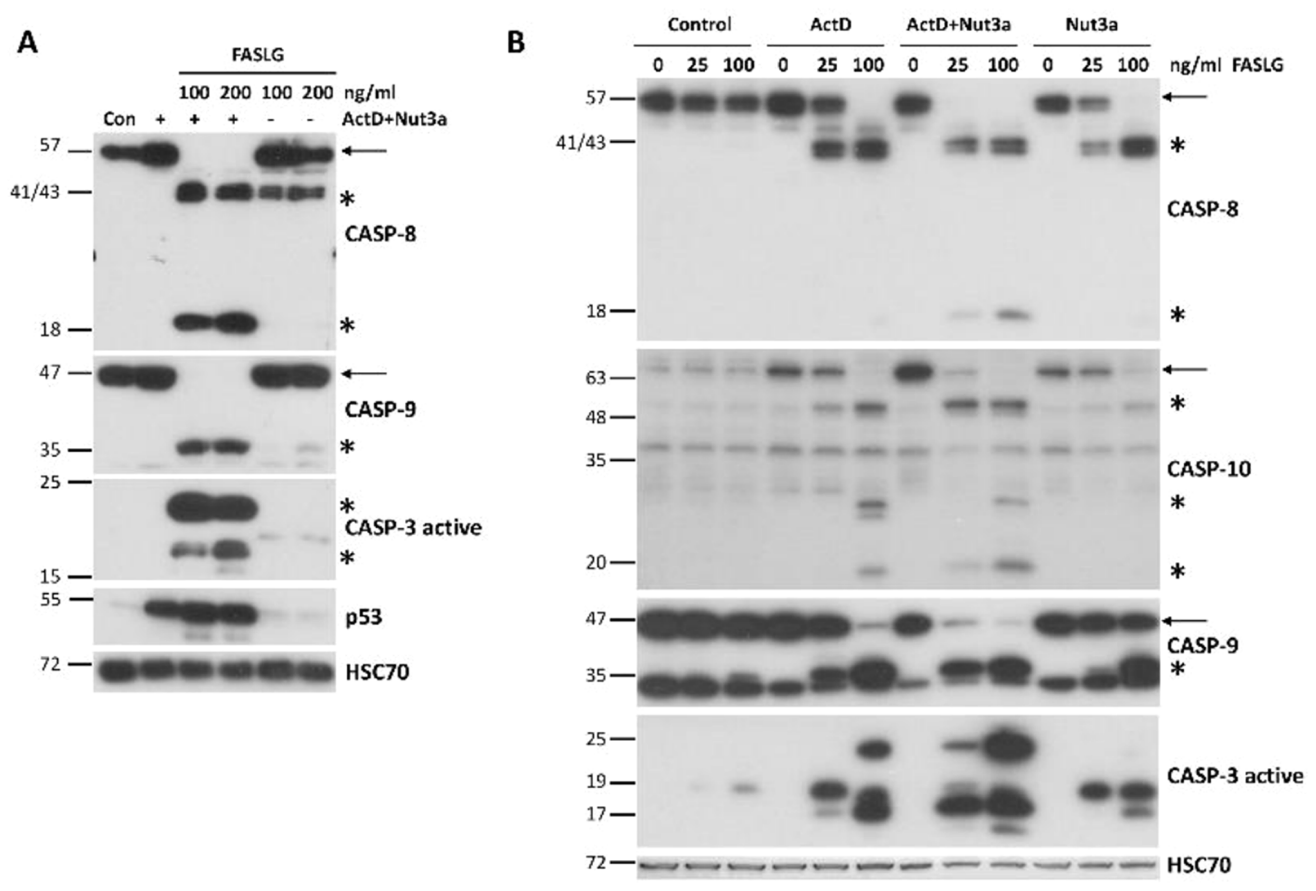
FASLG in A549 cells pre-exposed to actinomycin D and nutlin-3a activates caspases of extrinsic (caspase-8) and intrinsic (caspase-9) apoptotic pathways as well as of executioner caspase-3. (**A**) A549 cells were pre-exposed to actinomycin D and nutlin-3a (ActD + Nut3a) for 48 h and subsequently were treated for 2.5 h with FASLG in two concentrations. Control cells were mock-treated, while other cells were exposed only to ActD + Nut3a or FASLG. Throughout the paper, full-length caspases are marked by arrows and the cleaved forms of caspases are marked by asterisks. Left-side numbers represent protein sizes in kDa. **(B)** A549 cells were pre-exposed with actinomycin D (ActD), nutlin-3a (Nut3a), or both substances for 45 h and subsequently exposed for 2 h to FASLG at indicated concentrations. Some cells were only exposed to FASLG. Control cells were mock-treated. The expression of the indicated caspases was examined by Western blotting. HSC70 is the leading control

Most cancer cells are resistant to FAS-mediated apoptosis [9]. To find out if we could break this resistance, we pre-exposed A549 lung cancer cells to ActD and Nut3a for 48 h and then treated them for 2.5 h with FASLG at 100 or 200 ng/ml concentrations. As expected, a strong accumulation of p53 appeared, which is concomitant with its activation in cells exposed to ActD + Nut3a (Fig. 1A). In cells pre-exposed to ActD + Nut3a, FASLG induced splitting of caspase-8 (57 kDa) into cleaved intermediates (41 and 43 kDa) and an active fragment (18 kDa) (Fig. 1A). This was associated with the disappearance of full-length caspase-8, suggesting its activation in most cells. This implies that in the majority of cells, the apoptosis was initiated. To find out if the intrinsic pathway of apoptosis was also engaged, we examined the activation status of caspase-9. Only the cleaved product of caspase-9 (p35) was detected in cells pre-treated with ActD + Nut3a and exposed to FASLG, while no full-length protein (p47) was observed (Fig. 1A). This again suggests that apoptosis was initiated in the majority of cells and that the intrinsic apoptotic pathway was activated. We also observed the activation of caspase 3 (17 and 19 kDa fragments). The active caspase 3 appeared only in cells pre-exposed to ActD + Nut3a and treated with FASLG. Exposure of cells only to ActD + Nut3a did not result in detectable activation of either of the examined caspases. Exposure to FASLG alone induced only the appearance of a cleaved intermediate of caspase-8 (p41 and p43), indicating that the ligand-mediated apoptosis was initiated, but in most cells it did not reach the stage of activation of caspase-9 or caspase-3, which is consistent with the observations that cancer cells are generally resistant to FASLG-initiated apoptosis [9].

To find out whether the sensitization to FASLG-induced apoptosis results from the cooperation of ActD and Nut3a, we exposed A549 cells either separately to each compound or simultaneously to both of them. After pre-exposure, cells were treated for 2 h with FASLG at two concentrations (25 and 100 ng/ml). Subsequently, we examined the activation status of caspase-8, caspase-9, and caspase-3 (Fig. 1B). We observed strong cooperation between ActD and Nut3a in sensitizing the cells to FASLG. ActD or Nut3a acting separately sensitized cells to apoptosis induced by a 25 ng/ml concentration of FASLG, however, the level of caspase activation was small compared to ActD + Nut3a pre-treatment (compare the level of full-length caspases 8 and 9). The expression of activated caspase-3 was also higher in cells pre-exposed to ActD + Nut3a (Fig. 1B). Both actinomycin D and nutlin-3a acting separately sensitized the cells to a high concentration (100 ng/ml) of FASLG, but again, the effect was much stronger when both compounds acted together. Similar cooperation between actinomycin D and nutlin-3a in sensitizing cells to the pro-apoptotic effect of FASLG was observed in three other cell lines with wild-type p53: AGS, NCI-H460 and U-2 OS (Supplementary Figures S1 and S2).

Next, we examined the expression level of caspase-10 (Fig. 1B), which is a paralog of caspase-8, is also involved in the death receptor pathway of apoptosis [10] and was found in our transcriptomic data to be activated by ActD + Nut3a in various cancer cell lines [7]. Since the caspase-10 gene (*CASP10*) is activated by p53 [11], we hypothesized that treatment with ActD + Nut3a promotes the expression of this protein. Our data supported this hypothesis. Actinomycin D and nutlin-3a acting separately stimulated the expression of full-length caspase-10, but when combined, both substances acted in this regard at least additively (Fig. 1B). The addition of FASLG reduced the expression of full-length caspase-10 and resulted in the appearance of cleaved forms of approximately 50, 30, and 20 kDa. Regarding cleavage (activation) of caspase-10, actinomycin D and nutlin-3a acted cooperatively. FASLG at 25 ng/ml concentration did not reduce the level of full-length protein in cells pre-exposed to actinomycin D or nutlin-3a but in cells pre-exposed to ActD + Nut3a, the amount of full-length caspase-10 dropped significantly (Fig. 1B). Therefore, ActD + Nut3a promoted the expression of caspase-10, which was activated after exposure to FASLG.

**Fig. 2:**
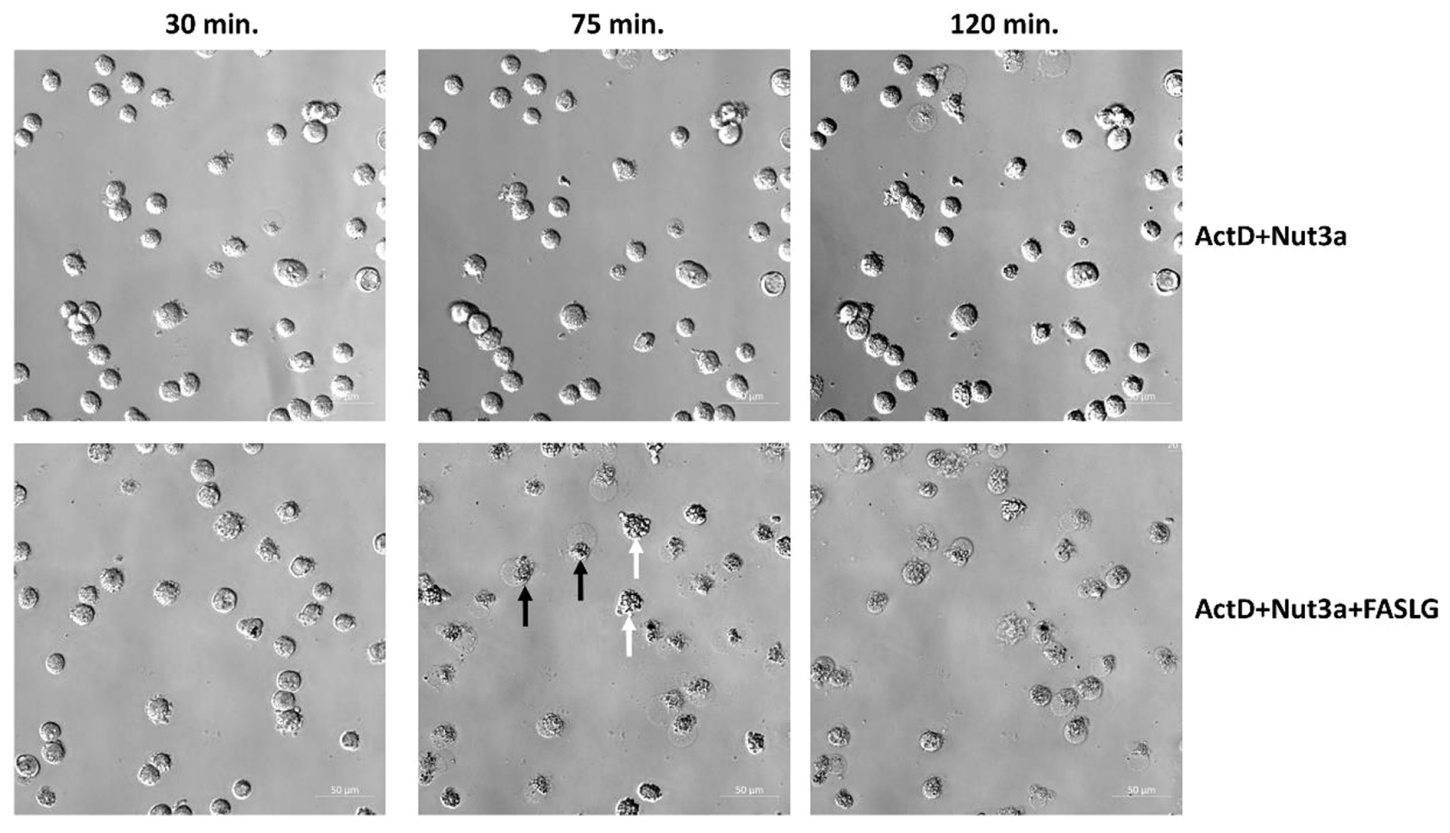
Microscope observations reveal frequent death of cells pre-exposed to actinomycin D and nutlin-3a and subsequently incubated with FASLG. A549 cells were pre-exposed to ActD + Nut3a for 45 h and subsequently incubated with or without 100 ng/ml FASLG. At the indicated times, the cells on the same fragment were photographed. White arrows mark examples of cells with early apoptotic morphology and black arrows mark cells with late apoptotic/necrotic morphology

To observe the morphology of cell death, we performed live-cell imaging. As shown in Fig. 2, after 75 min. of incubation with FASLG, most cells pre-exposed to ActD + Nut3a show either signs of early apoptosis or late apoptosis/necrosis. In the cell population pre-exposed to ActD + Nut3a, and subsequently growing in control conditions, cell death was infrequent even after 120 min. This simple observation indicates that the cells pre-exposed to ActD + Nut3a and treated with FASLG die quickly and the death is observed in the majority of cells. Similar conclusions can be drawn from live-cell imaging of U-2 OS cells (supplementary figure, Fig. S3).

**Fig. 3:**
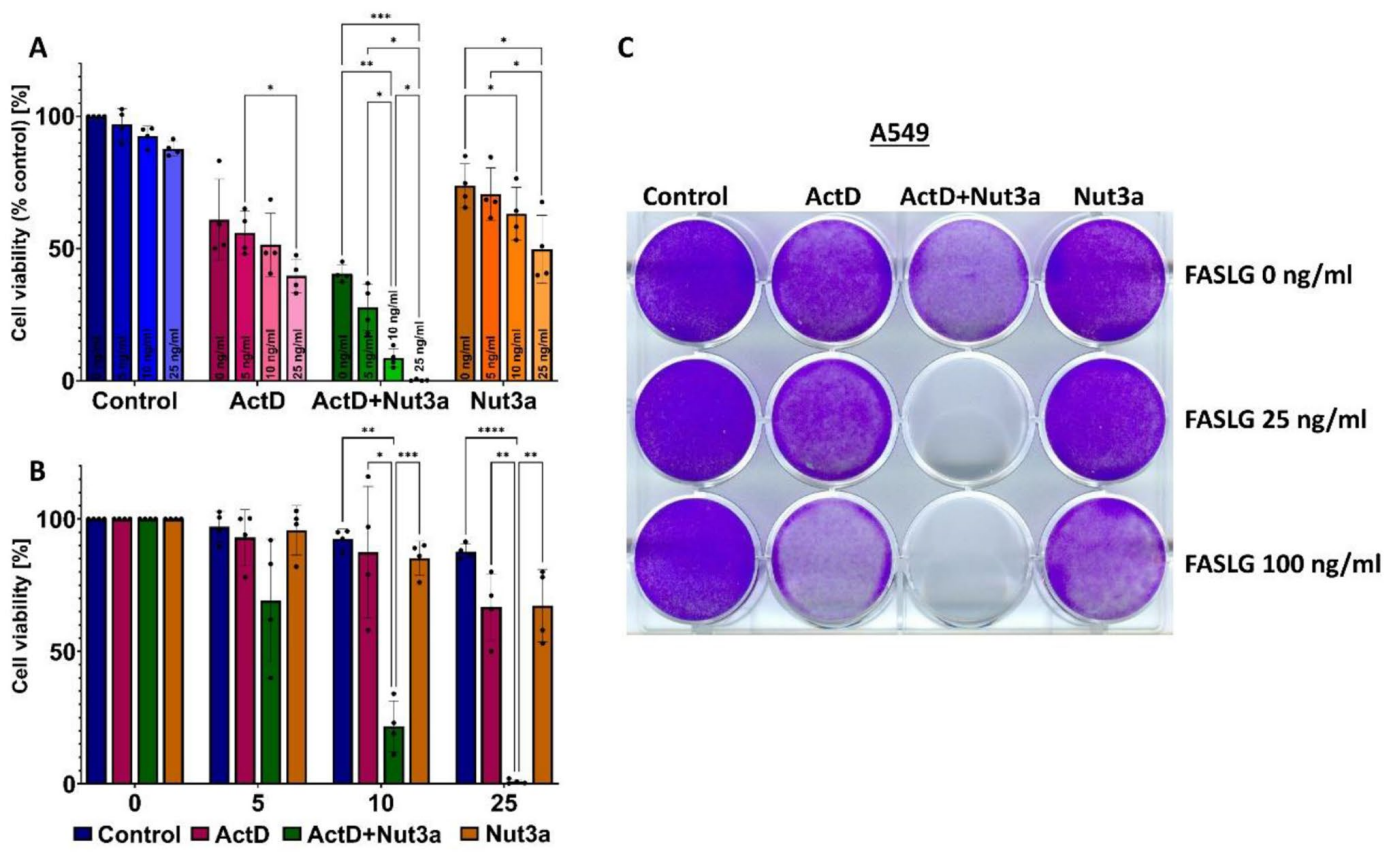
Actinomycin D and nutlin-3a collaborate in sensitizing A549 cells to cell death induced by FASLG. (A) The relative cell viability measured by MTS assay. The viability of cells treated neither with drugs nor with FASLG was set to 100%. **(B)** The data from Graph **A** presented in a modified fashion. The viability of mock-treated cells or cells exposed to any drag without subsequent exposure to FASLG was set to 100%. This allows for visualizing the effect of FASLG alone (at indicated concentrations ng/ml) on cell viability. The statistical significance of the differences was calculated by two-way ANOVA and Tukey’s multiple comparisons test (*P ≤ 0.05, **P ≤ 0.01, ***P ≤ 0.001, ****P ≤ 0.0001). **(C)** Cells attached to the wells of the culture plate (stained by crystal violet) following the indicated treatment regimen. The cells were pretreated with drugs for 46 h and subsequently left untreated (the top row) or they were exposed to the indicated concentrations of FASLG for 5 h. Surviving cells were allowed to recover for 70 h before fixation and staining

Next, we used the MTS assay to measure the viability of the cells (Fig. 3A). The A549 cells were pre-exposed for 45 h and subsequently treated for 5 h with increasing concentrations of FASLG (5, 10, and 25 ng/ml). FASLG acting on untreated cells only marginally reduced their viability (88% of the control for 25 ng/ml). When cells were pre-exposed to ActD + Nut3a and subsequently treated with 25 ng/ml FASLG, their viability dropped to less than 1% of control. Thus, consistent with the Western blotting and microscopy observations, pretreatment with ActD + Nut3a and subsequent exposure to FASLG with at least 25 ng/ml concentration killed most cells. To better illustrate the influence of FASLG, we transformed this graph by setting the viability of cells not exposed to FASLG (control, ActD, ActD + Nut3a, or Nut3a) to 100% (Fig. 3B). The effect of collaboration between ActD and Nut3a on the sensitization to FASLG was best-visible at 25 ng/ml concentration. When cells were pre-exposed either to actinomycin D or nutlin-3a, the addition of FASLG reduced viability to approximately 65%, whereas when two compounds were combined, FASLG reduced cell viability to 0.8%. To visualize this effect macroscopically, the experiment was performed with cells seeded onto a 12-well plate. The results are presented in Fig. 3C. In the columns, cells were either mock-treated or exposed (45 h) as shown. Subsequently, in the rows, the cells were exposed to FASLG (25 and 100 concentration) for 5 h or were left untreated (the top row). After treatment, the cells were left for 70 h in fresh medium to allow the surviving cells to regrow. The staining revealed that on the plate pre-exposed either to actinomycin D or nutlin-3a and treated with FASLG (25 ng/ml) the cells reached confluence after recovery, while the cells in the well exposed to ActD + Nut3a and FASLG did not regrow (no visible staining). Thus, the overwhelming majority of cells pretreated with ActD + Nut3a and exposed to FASLG at 25 ng/ml concentration did not survive the treatment. A similar effect was observed in other cell lines with wild-type p53 - U-2 OS and NCI-H460 (Supplementary Figure S4). Another assay helped to estimate that FASLG reduced the clonogenic potential of A549 cells exposed to ActD + Nut3a by a factor of roughly 1000 (Fig. S5).

**Fig. 4:**
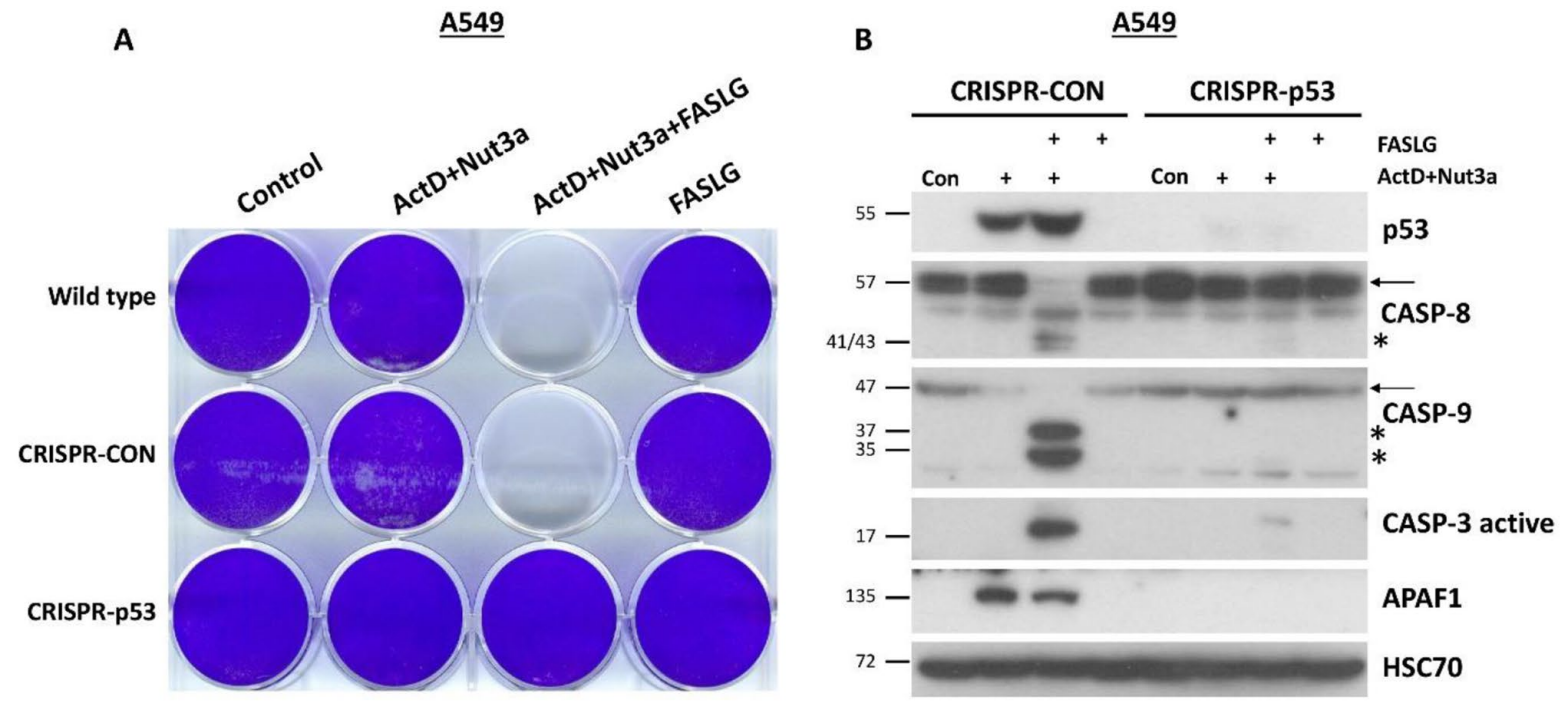
p53 is indispensable for the induction of apoptosis of cells pre-exposed to actinomycin D and nutlin-3a. (**A**) The stained cells on the culture plate. The cells of the parental cell line (A549, wild-type), controls for knockdown (CRISPR-CON), and the p53-deficient cells (CRISPR-p53) were pre-exposed as indicated for 45 h and subsequently treated with FASLG and 100 ng/ml concentration for 5 h. The surviving cells were allowed to recover for 72 h. The cells were allowed to overgrow in order to better visualize the scarcity of cells on the wells exposed to ActD + Nut3a + FASLG. **(B)** The expression of indicated proteins as detected by Western blotting. The p53-proficient (CRISPR-CON) and deficient cells (CRISPR-p53) were exposed to ActD + Nut3a for 45 h followed by exposure to FASLG at 50 ng/ml concentration for 2.5 h

To find out if the caspase activity is indispensable for the cell death induced by our treatment method, we employed the caspase inhibitors (Fig. S6). One of them was Z-VAD-FMK, which inhibits all caspases and the other was Z-IETD-FMK, which specifically inhibits caspase-8. When the inhibitors were employed one hour before exposure to FASLG we observed the inhibition but not total block of activation of both caspase-8 and caspase-9 (Fig. S6 A). The Z-VAD-FMK inhibitor almost totally blocked the activation of caspase-8 in Jurkat cells exposed only to etoposide (Fig. S6 B). When we applied the inhibitors in the MTS assay, we noticed that the FASLG-induced cell death was diminished but not totally blocked (Fig. S6 C). Bases on these observations, we conclude that even the high concentration of inhibitors (100 µM) was not able to prevent totally the cell death induced by our treatment method, either because some caspase-independent mechanisms are at play or because the induced activity of caspases is so high that the commonly used inhibitors are unable to stop it.

Then, we tested if p53 participates in the sensitization. We started by using the method of cell staining on a 12-well plate. We used parental A549 cells, A549 cells with the expression of p53 knocked down by CRISPR/Cas9 technology, and the controls for knockdown created previously [8]. These cells were seeded in the rows of the plate (Fig. 4A). The cells in columns were mock-treated or exposed to ActD + Nut3a, ActD + Nut3a + FASLG, or FASLG. After the recovery, no cell staining was visible in the wells of parental cells or controls exposed to ActD + Nut3a + FASLG, whereas the p53 knockdown cells reached the confluence. Therefore, the sensitizing effect of ActD + Nut3a was strictly dependent on the presence of wild-type p53. Next, we performed a similar experiment; however, the treatment time with FASLG was shorter (2.5 h) and cells were collected directly after treatment for cell lysate preparation. The Western blot presented in Fig. 4B shows that the activation of caspases − 8, -9, and − 3 was detectable only in control cells for knockdown. The APAF1 is a protein coded by the p53-regulated gene, which is involved in apoptosome formation and activation of caspase-9 [3, 12]. The lack of accumulation of APAF1 in p53 knockdown cells exposed to ActD + Nut3a confirms that they have no functional p53. The experiment carried out in NCI-H460 cells with p53 expression knocked down by CRISPR/Cas9 technology using the same method described for A549 cells [8] yielded similar results (Supplementary Figure S7).

**Fig. 5:**
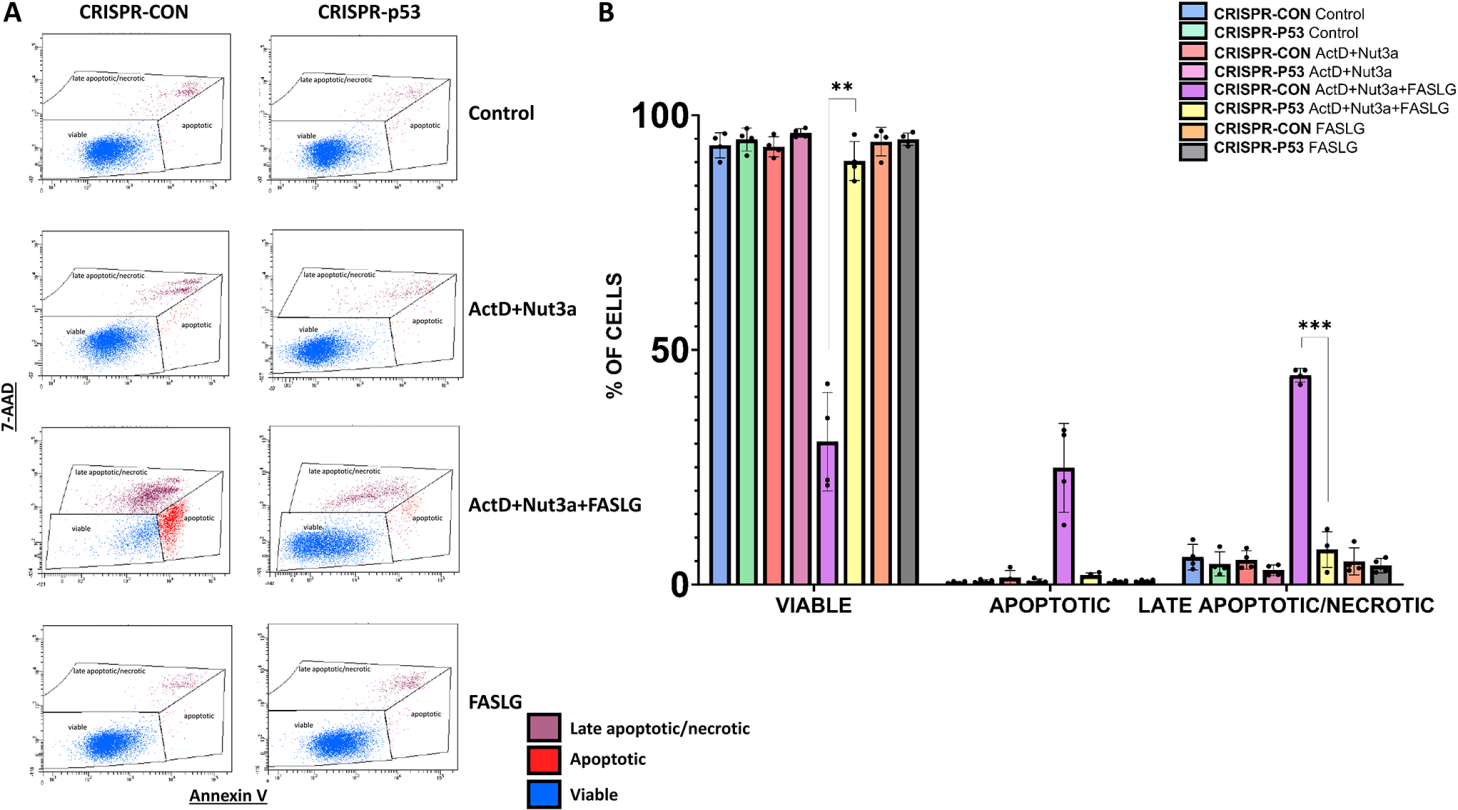
Flow cytometry showing apoptosis induced by FASLG in p53-proficient cells pre-exposed to actinomycin D and nutlin-3a. (**A**) An example of various cell populations (viable, early apoptotic, late apoptotic/necrotic) detected by cytometric analysis of cells stained for Annexin V and 7-AAD. The cells were treated as indicated. The pre-exposure time to ActD + Nut3a was 45 h, exposure time to FASLG at 50 ng/ml was 2.5 h. **(B)** The frequency of the indicated cell populations of p53-proficient and p53-deficient A549 cells measured in four biological replicates. The statistical significance of the differences was calculated using the Brown-Forsythe ANOVA test and Welch ANOVA test, Dunnett’s T3 multiple comparisons test, **P ≤ 0.01, ***P ≤ 0.001

Subsequently, we measured the frequency of apoptotic cells by flow cytometry. The raw data for one biological replicate are presented in Fig. 5A and the graph shows the data from four biological replicates (Fig. 5B). Apoptotic cells were very rare after exposure to ActD + Nut3a. The frequency of both early apoptotic cells (high staining for Annexin V and low staining for 7-AAD) and late apoptotic/necrotic cells (high staining with 7-AAD) increased strongly in cells pre-exposed to ActD + Nut3a and treated with FASLG, but only in the case of p53-proficient cells. Consequently, the frequency of viable p53-proficient cells exposed to ActD + Nut3a + FASLG was significantly lower than in the case of p53-deficient cells. This experiment further confirms that p53 is required to induce apoptosis in cells exposed to ActD + Nut3a + FASLG. In this experiment, we have more viable cells than in the MTS test because, for cytometric analysis, the cells were exposed to FASLG for only 2.5 h.

**Fig. 6:**
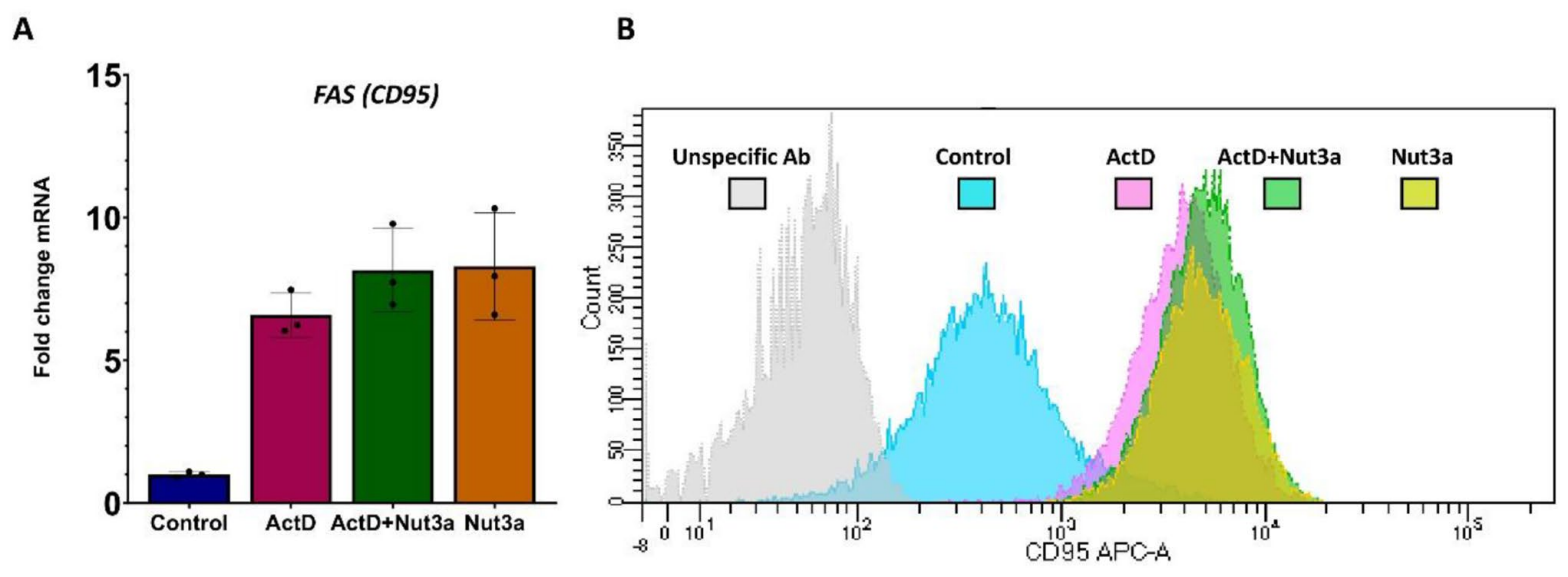
Actinomycin D and nutlin-3a do not collaborate in increasing the expression of FAS mRNA or FAS protein on the cell surface. (**A**) The expression of FAS mRNA measured by RNA-Seq in three biological replicates for treatment of A549 cells with ActD (5nM), Nut3a (5 µM), or ActD + Nut3a for 30 h. The raw RNA-Seq data were published [7] and deposited in the Sequence Read Archive (SRA) under accession number PRJNA831359. **(B)** The expression of the FAS receptor (CD95) measured by flow cytometry of A549 cells treated as indicated and stained with anti-CD95 (FAS) antibody. The cells were exposed to the indicated compounds for 48 h

What is the mechanism used by p53 to overcome the resistance of cells to FASLG? The data presented on the Western blots (Fig. 4B) indicate that p53-deficient cells are not able to activate caspase-8, thus the defect is already present at the beginning of the signaling pathway, which starts with the FAS receptor. Hence, the logical conclusion is that the combination of ActD + Nut3a stimulates the expression of the *FAS* gene. Our transcriptomic data show that this is the case (Fig. 6A). However, the collaboration of actinomycin D and nutlin-3a in sensitizing cells to FASLG-dependent apoptosis cannot arise from the activation of the *FAS* gene because these substances do not cooperate in its expression (Fig. 6A). The other possibility is that actinomycin D and nutlin-3a collaborate in augmenting the expression of the FAS receptor on the plasma membrane. However, the cytometric measurement of FAS on the cell surface showed similar expression under all treatment conditions (Fig. 6B).

One of the proteins located on the top of the signaling pathway starting from death receptors is the aforementioned caspase-10 [13]. *The CASP10* gene is activated by p53 [11]. Therefore, by up-regulating caspase-10, p53 can also promote the extrinsic apoptotic pathway. We demonstrated that actinomycin D and nutlin-3a cooperate in the upregulation of caspase-10 in A549 cells (Fig. 1B). To find out if a similar effect occurs in other cell types, we performed the treatment of various cell lines with wild-type p53. The data are shown in Fig. 6A. In all four cell lines, treatment with ActD + Nut3a leads to the accumulation of caspase-10. Moreover, in three cell lines, actinomycin D and nutlin-3a do not collaborate in the upregulation of the FAS receptor (Fig. 6A). We also examined the expression of caspase-6, which is coded by the gene (*CASP6*) activated by ActD + Nut3a as shown by our transcriptomic data [7, 8]. Caspase-6 is an executioner caspase [3] but it can also directly activate caspase-8 following activation of the mitochondrial pathway of apoptosis [14], which can create a positive feedback loop. Furthermore, *CASP6* is directly activated by p53 [15]. In three cell lines, the amount of caspase-6 increased following exposure to ActD + Nut3a (Fig. 6A). Thus, the upregulation of *CASP6* by treatment with ActD + Nut3a must be considered as a plausible element of increased sensitivity of cells to FASLG.

**Fig. 7:**
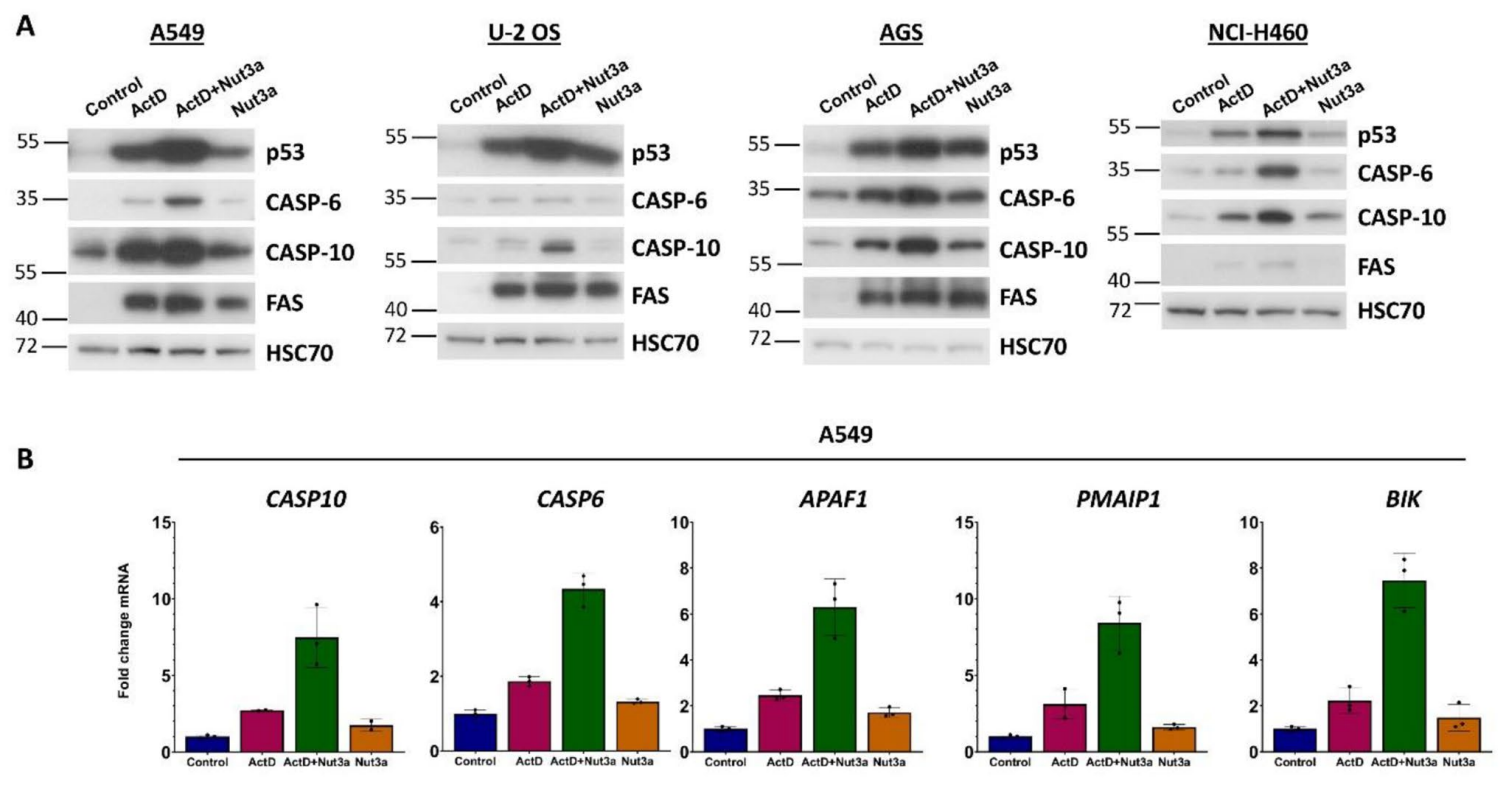
Actinomycin D and nutlin-3a collaborate in the activation of selected p53-regulated, pro-apoptotic genes. (**A**) Western blotting showing the expression of the p53, caspase-6, caspase-10, and FAS receptor in indicated cell lines exposed as shown for 48 h. **(B)** The expression of the indicated genes, as measured by the aforementioned RNA-Seq, in A549 cells growing in control conditions or exposed to the indicated substances for 30 h [7]

Our transcriptomic data demonstrated that in A549 cells, actinomycin D and nutlin-3a cooperate in the activation of *CASP10* and *CASP6* (Fig. 7B), which is consistent with our Western blotting results (Fig. 7A). Other proteins may also contribute to the sensitizing effect of actinomycin D and nutlin-3a because these substances collaborate (Fig. 7B) in the activation of other p53-regulated, pro-apoptotic genes like *APAF1* [12], *PMAIP1* [16], and *BIK* [17].

**Fig. 8:**
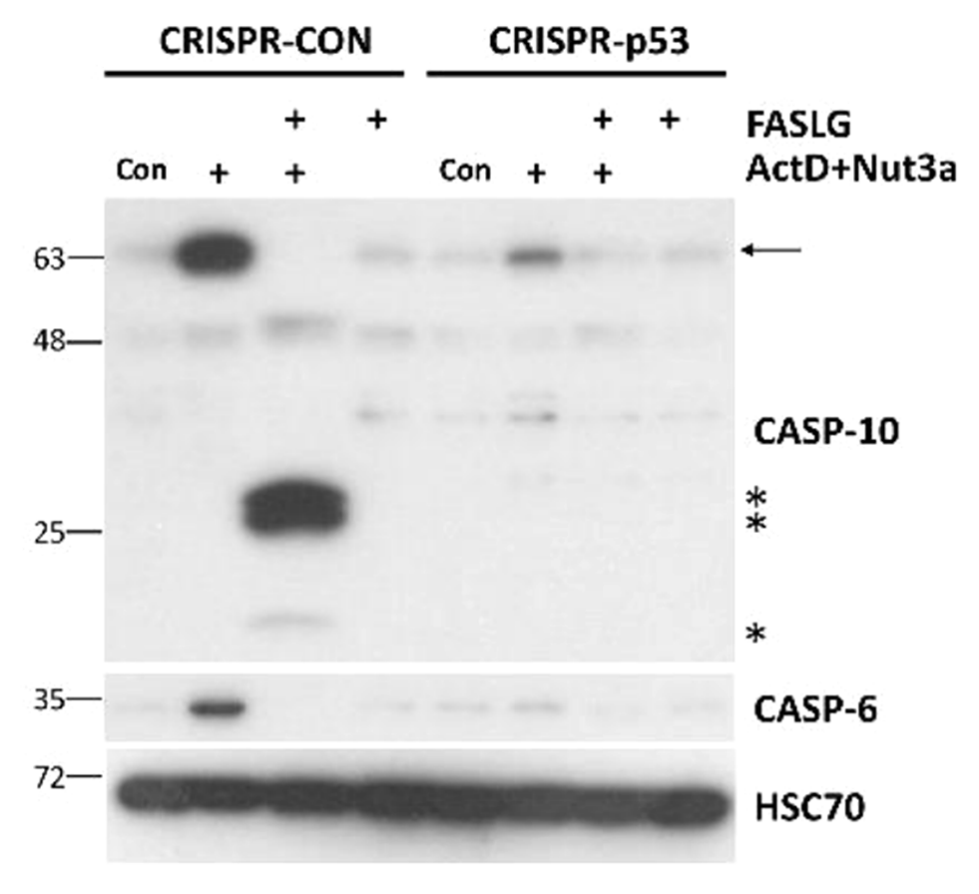
The accumulation of caspase-6 and caspase-10 following treatment with actinomycin D and nutlin-3a does not occur in p53-deficient cells. Western blotting showing the expression of caspase-10 (full-length and cleaved form) and caspase-6 (full-length form) in p53-proficient and p53-deficient cells treated as described in Fig. 4B

Regulation of *CASP6* and *CASP10* by p53, as mentioned above, was detected by others [11, 15], however, it was not extensively studied. Our Western blotting performed on lysates from p53-proficient and p53-deficient A549 cells (Fig. 8) confirms that these two genes are activated in a p53-dependent manner. It should be noted that, similarly to caspase-10, the full-length caspase-6 disappears in cells exposed to FASLG, probably due to activation by cleavage, although we could not detect the cleaved form on our blots.

**Fig. 9:**
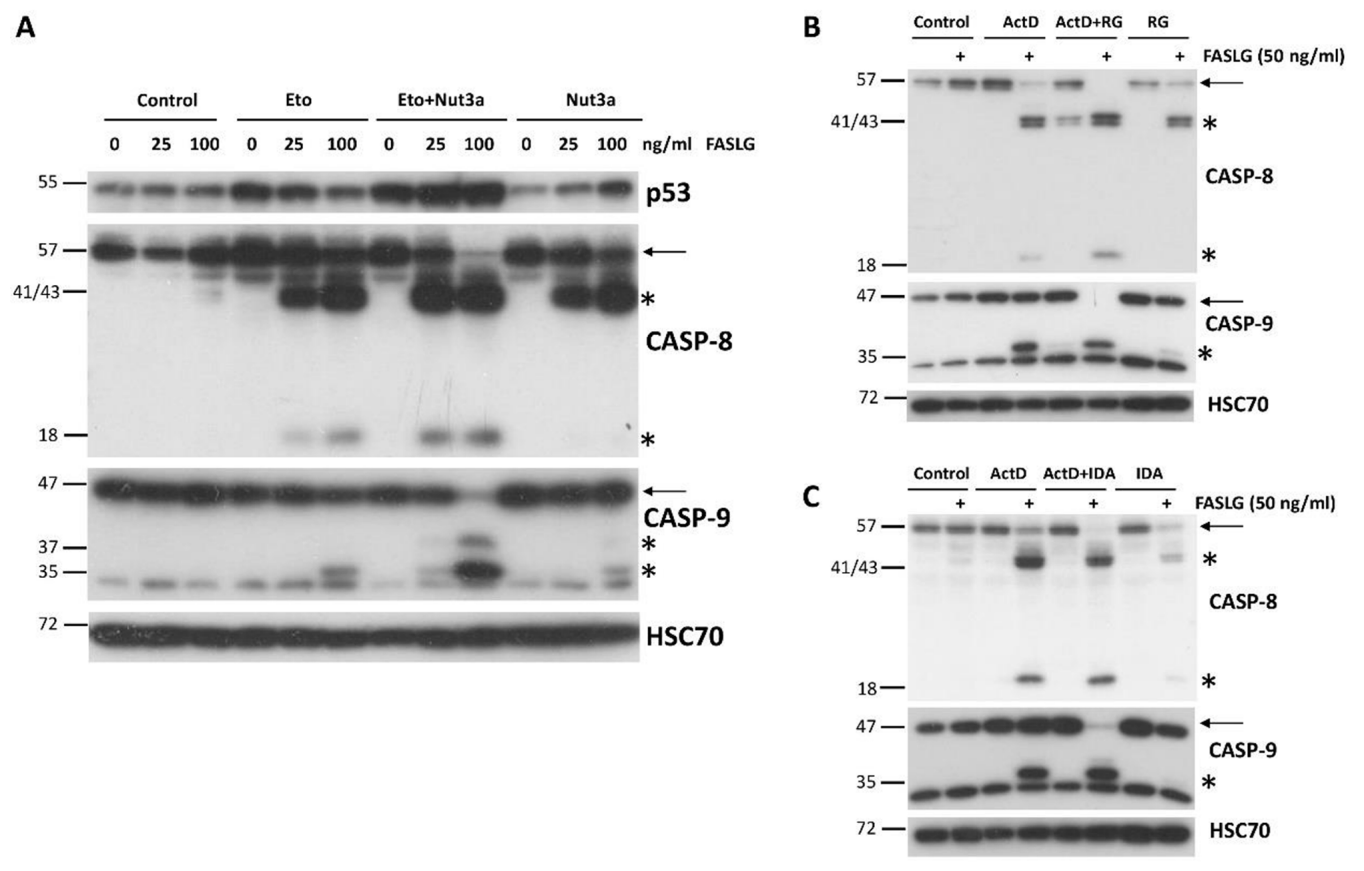
Drugs other than actinomycin D and nutlin-3a can collaborate in sensitizing cells to the pro-apoptotic activity of FASLG. (**A**) A549 cells were pre-exposed to etopiside (Eto; 5 µM), nutlin-3a, and their combination with subsequent exposure to FASLG for 2.5 h. The expression of p53 and the indicated caspases was examined by Western blotting. **(B)** A549 cells were pre-exposed to actinomycin D, RG7112 (RG), or both substances (A + RG), and then treated for 2.5 h with 50 ng/ml FASLG. Western blotting shows the expression of the indicated proteins. **(C)** A similar experiment as in B was performed with Idasanutlin (IDA) in place of RG7112 as an antagonist of MDM2

The next experiments were designed to find out if actinomycin D and nutlin-3a can be substituted by other compounds to reach a similar proapoptotic effect. First, we substituted actinomycin D with etoposide (Eto), which can also activate p53 [18]. The Western blot shows a clear collaboration between Eto and nutlin-3a (Fig. 9A). Moreover, we substituted nutlin-3a with other antagonists with p53-MDM2 interaction, idasanutlin, or RG7112 [19, 20]. Their co-incubation with actinomycin D also strongly sensitized the A549 cells to the pro-apoptotic activity of FASLG (Fig. 9B).

**Fig. 10:**
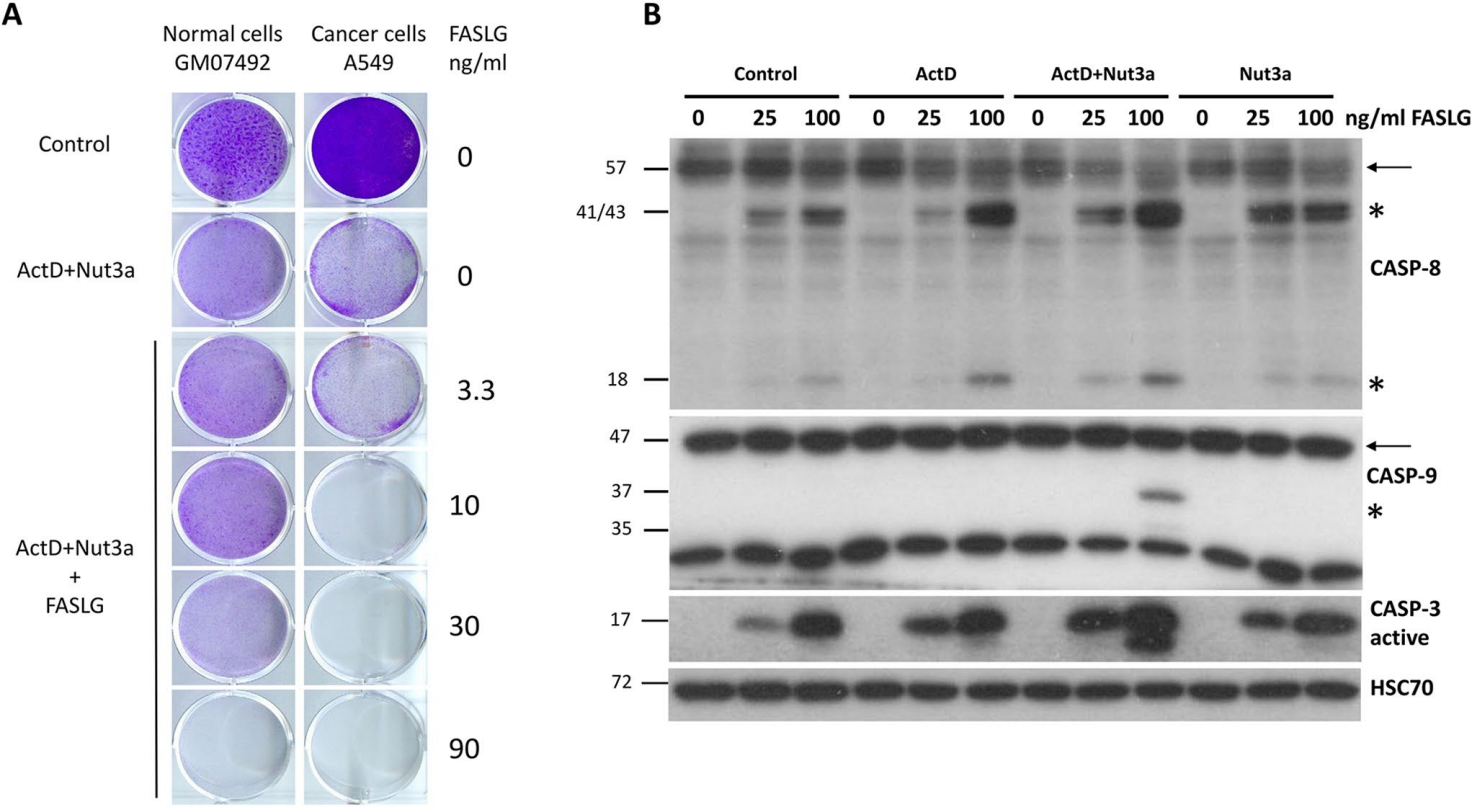
Normal human fibroblasts are less sensitive than cancer cells (A549) to the cytotoxic effect of ActD + Nut3a + FASLG treatment. (**A**) An equal number of early passage normal human fibroblasts were seeded as indicated on the wells of a 12-well plate. Subsequently, the cells were mock-treated (control) or exposed to ActD + Nut3a for 45 h. The cells were exposed to increasing concentrations of FASLG as indicated. The cancer cells (A549) were seeded and treated analogously. Both cell types were grown in the same medium (DMEM + 15% FBS) and were exposed (5 h) to the same preparation of FASLG in the medium. After treatment, the cells were allowed to recover for 90 h before fixation and staining. The original picture of the 12-well plate was cut, and the wells were arranged in a way that helped analyze the results. **(B)** Normal human fibroblasts were treated as indicated (ActD, Nut3a, or their combination for 45 h, FASLG for 2.5 h) and the expression of caspases was detected by Western blotting

To find out how noncancerous cells react to the treatment with ActD + Nut3a + FASLG, we compared how normal human fibroblasts (GM07492) and lung cancer cells (A549) grow after pre-treatment with ActD + Nut3a with subsequent exposure to FASLG (Fig. 10A). Using cell staining, we noticed that cancer cells were more susceptible to death at the intermediate concentrations of FASLG (10 and 30 ng/ml). We could not perform the viability assay because we observed that these fibroblasts poorly metabolized the MTS used for the test. Western blotting of the cells treated as shown in Fig. 10B, analogously to the treatment of cancer cells presented in Fig. 1B, revealed that there is no strong sensitizing effect of the ActD + Nut3a combination on FASLG-induced apoptosis. ActD + Nut3a pretreatment enables FASLG to activate caspase-9 but only when the ligand is applied at a high concentration (100 ng/ml). Moreover, even in these conditions, the activation of caspase-9 was not very strong. The activation of caspase-3 was very similar after the addition of FASLG regardless of earlier pre-treatment. Because, in these fibroblasts, caspase-3 can be activated by FASLG without caspase-9 activation, these cells apparently belong to the type that can directly activate caspase-3 by active caspase-8.

## Discussion

In this report, we demonstrated that actinomycin D and nutlin-3a cooperate to sensitize cancer cells to apoptosis induced by FASLG. This finding may have therapeutic implications. Shortly after the discovery of the pro-apoptotic activity of FASLG, it was considered to be a candidate for an anticancer drug. However, an unexpected major obstacle appeared, namely, the FAS agonist antibodies induced the death of hepatocytes leading to fulminant hepatitis and the death of treated animals [21]. In principle, our method of sensitization of cancer cells to FAS agonists creates an opportunity to use them at lower doses. Experiments with modified FASLG performed by others suggest that preventing hepatotoxicity is feasible [22, 23]. Moreover, our observations may improve the therapeutic application of the antagonists of MDM2-p53 interaction like nutlin-3a, idasanutlin, RG7112, and many others. Their therapeutic use is limited by their inefficiency in killing cancer cells – they mainly induce cell-cycle arrest [24]. However, combined with actinomycin D (or etoposide or probably other p53 activators), the antagonists of MDM2 can sensitize cancer cells to the destruction by a patient’s own cytotoxic lymphocytes or natural killer cells, which employ FASLG on their surface to induce the death of target cells [25].

The mechanism of sensitization of cancer cells to FASLG by actinomycin D and nutlin-3a, or similar drug combinations, is not known but depends strictly on wild-type p53. Apparently, there is something extraordinary about the set of genes regulated by p53 in cells exposed to ActD + Nut3a; some of these genes make cancer cells extremely sensitive to FASLG. The most obvious hypothesis was that actinomycin D and nutlin-3a interactively increase the expression of the FAS receptor. However, the analysis of its mRNA level, the total amount of FAS protein, or its expression on the cell surface did not support this hypothesis (Figs. 6 and 7). We noticed that actinomycin D and nutlin-3a cooperate at least additively in the activation of the *CASP10* gene, which encodes caspase-10, the protein, which, like caspase-8, also conveys the pro-apoptotic signal from the FAS receptor to executioner caspases [26]. However, at least one report indicates that in some conditions caspase-10 inhibits FASLG-induced apoptosis by impeding DISC-mediated caspase-8 activation. The study demonstrated that caspase-10 promotes FASLG-mediated activation of the NF-κB transcription factor and gene induction [27]. These authors used cells with the constitutive expression of caspase-10, which was deliberately downregulated, resulting in increased apoptosis. In our model, caspase-10 was strongly upregulated by A + N (Figs. 7 and 8). Thus, the function of this protein in the regulation of apoptosis triggered by death receptors is controversial. It is plausible that the role that this protein plays in the regulation of apoptosis depends on its expression level and may even be antiapoptotic when the level is low and pro-apoptotic when it is strongly upregulated. This is, of course, pure speculation, but our method of powerful and fast induction of apoptosis, which is associated with upregulation and cleavage of caspase-10, may be used to resolve this controversy.

In addition to *CASP10*, actinomycin D and nutlin-3a cooperate in the activation of other p53-regulated, pro-apoptotic genes such as *CASP6*, *APAF1*, *PMAIP1*, *BIK*, and many others [7, 8]. Thus, it is very likely that there is no single p53 target gene, which is a crucial element of this sensitizing mechanism. Probably, every p53 target gene related to apoptosis contributes partially to the sensitization, however, this hypothesis requires further testing. Our recently published transcriptomic data on various cell lines exposed to ActD + Nut3a [7] may help to generate such hypotheses. For example, it is plausible that the products of some genes activated by p53 modify the physicochemical properties of cell membranes, which can promote the formation of the membrane rafts that are essential for the initiation of FAS-induced cell death [28]. Thus, the mechanisms of p53-dependent sensitization of cells to apoptosis may not always be overt.

Our results clearly indicate that even strong activation of many p53-regulated, pro-apoptotic genes may not be sufficient to induce apoptosis as long as a crucial trigger is missing. In the case of cells with p53 activated by ActD + Nut3a, this trigger is FASLG. We speculate that treatment with ActD + Nut3a resembles a naturally occurring stress factor, which activates p53 in a similar fashion and leads to a strong response to FASLG. Considering that infected cells are physiological targets of FASLG-bearing lymphocytes, we hypothesize that treatment with ActD + Nut3a resembles stress within the infected cells, which activates p53 and makes them susceptible to FASLG.

Unexpectedly, it was found that the FAS ligand can, in some conditions, promote tumor development. For instance, FASLG-containing microvesicles derived from cancer cells can kill T lymphocytes [29]. Thus, tumor cells can use FASLG to suppress the attack by the immune system. Furthermore, the FAS receptor can engage non-apoptotic, growth-promoting pathways [30]. Unexpectedly, some cancer cells depend on the constitutive activity of FAS, stimulated by cancer-produced FASLG, for optimal growth [31]. The microenvironment of some cancers appears to be permeated with a high level of FASLG [32, 33]. If this is the case, treatment with ActD + Nut3a can change FASLG from a promoter of cancer growth to its inhibitor. Our observations clearly point toward novel approaches to cancer therapy employing the pro-apoptotic pathway starting from the FAS ligand or other agonists of death receptors. Moreover, they support the notion that p53 is an important element of immunity [34].

## Electronic supplementary material

Below is the link to the electronic supplementary material.

Supplementary Material 1

## Data Availability

No datasets were generated or analysed during the current study.

## References

[1] Aubrey BJ, Kelly GL, Janic A, Herold MJ, Strasser A (2018) How does p53 induce apoptosis and how does this relate to p53-mediated tumor suppression? Cell Death Differ 25(1):104–113. 10.1038/cdd.2017.169 29149101 10.1038/cdd.2017.169 PMC5729529

[2] Fouqué A, Debure L, Legembre P (2014) The CD95/CD95L signaling pathway: a role in carcinogenesis. Biochim Biophys Acta 1846(1):130–141. 10.1016/j.bbcan.2014.04.007 24780723 10.1016/j.bbcan.2014.04.007

[3] Green DR, Llambi F, Cell Death S (2015) Cold Spring Harb Perspect Biol 1;7(12):a006080. 10.1101/cshperspect.a006080 10.1101/cshperspect.a006080 PMC4665079 26626938

[4] Müller M, Wilder S, Bannasch D, Israeli D, Lehlbach K, Li-Weber M, Friedman SL, Galle PR, Stremmel W, Oren M, Krammer PH (1998) p53 activates the CD95 (APO-1/Fas) gene in response to DNA damage by anticancer drugs. J Exp Med 188(11):2033–2045. 10.1084/jem.188.11.2033 9841917 10.1084/jem.188.11.2033 PMC2212386

[5] Micheau O, Solary E, Hammann A, Martin F, Dimanche-Boitrel MT (1997) Sensitization of cancer cells treated with cytotoxic drugs to fas-mediated cytotoxicity. J Natl Cancer Inst 89(11):783–789. 10.1093/jnci/89.11.783 9182976 10.1093/jnci/89.11.783

[6] Zajkowicz A, Gdowicz-Kłosok A, Krześniak M, Ścieglińska D, Rusin M (2015) Actinomycin D and nutlin-3a synergistically promote phosphorylation of p53 on serine 46 in cancer cell lines of different origin. Cell Signal 27(9):1677–1687. 10.1016/j.cellsig.2015.05.005 25989210 10.1016/j.cellsig.2015.05.005

[7] Łasut-Szyszka B, Gdowicz-Kłosok A, Małachowska B, Krześniak M, Będzińska A, Gawin M, Pietrowska M, Rusin M (2024) Transcriptomic and proteomic study of cancer cell lines exposed to actinomycin D and nutlin-3a reveals numerous novel candidates for p53-regulated genes. Chem Biol Interact 392:110946. 10.1016/j.cbi.2024.110946 38460933 10.1016/j.cbi.2024.110946

[8] Łasut-Szyszka B, Małachowska B, Gdowicz-Kłosok A, Krześniak M, Głowala-Kosińska M, Zajkowicz A, Rusin M (2021) Transcriptome Analysis of Cells exposed to actinomycin D and Nutlin-3a reveals new candidate p53-Target genes and indicates that CHIR-98014 is an important inhibitor of p53 activity. Int J Mol Sci 22(20):11072. 10.3390/ijms222011072 34681730 10.3390/ijms222011072 PMC8538697

[9] Peter ME, Hadji A, Murmann AE, Brockway S, Putzbach W, Pattanayak A, Ceppi P (2015) The role of CD95 and CD95 ligand in cancer. Cell Death Differ 22(4):549–559. 10.1038/cdd.2015.3 25656654 10.1038/cdd.2015.3 PMC4356349

[10] Sprick MR, Rieser E, Stahl H, Grosse-Wilde A, Weigand MA, Walczak H (2002) Caspase-10 is recruited to and activated at the native TRAIL and CD95 death-inducing signaling complexes in a FADD-dependent manner but cannot functionally substitute caspase-8. EMBO J 21(17):4520–4530. 10.1093/emboj/cdf441 12198154 10.1093/emboj/cdf441 PMC126181

[11] Rikhof B, Corn PG, El-Deiry WS (2003) Caspase 10 levels are increased following DNA damage in a p53-dependent manner. Cancer Biol Ther 2(6):707–712 PMID: 14688482 14688482

[12] Robles AI, Bemmels NA, Foraker AB, Harris CC (2001) APAF-1 is a transcriptional target of p53 in DNA damage-induced apoptosis. Cancer Res61(18):6660-4. PMID: 11559530 11559530

[13] Wang J, Chun HJ, Wong W, Spencer DM, Lenardo MJ (2001) Caspase-10 is an initiator caspase in death receptor signaling. Proc Natl Acad Sci U S A98(24):13884–13888. 10.1073/pnas.241358198 10.1073/pnas.241358198 PMC61136 11717445

[14] Cowling V, Downward J Caspase-6 is the direct activator of caspase-8 in the cytochrome c-induced apoptosis pathway: absolute requirement for removal of caspase-6 prodomain (2002) cell death Differ9(10):1046–1056. 10.1038/sj.cdd.4401065 10.1038/sj.cdd.4401065 12232792

[15] MacLachlan TK, El-Deiry WS (2002) Apoptotic threshold is lowered by p53 transactivation of caspase-6. Proc Natl Acad Sci U S A99(14):9492–9497. 10.1073/pnas.132241599 10.1073/pnas.132241599 PMC123168 12089322

[16] Hudson CD, Morris PJ, Latchman DS, Budhram-Mahadeo VS (2005) Brn-3a transcription factor blocks p53-mediated activation of proapoptotic target genes Noxa and Bax in vitro and in vivo to determine cell fate. J Biol Chem 25(12):11851–11858. 10.1074/jbc.M408679200 10.1074/jbc.M408679200 15598651

[17] Mathai JP, Germain M, Marcellus RC, Shore GC (2002) Induction and endoplasmic reticulum location of BIK/NBK in response to apoptotic signaling by E1A and p53. Oncogene 21(16):2534–2544. 10.1038/sj.onc.1205340 11971188 10.1038/sj.onc.1205340

[18] Karpinich NO, Tafani M, Rothman RJ, Russo MA, Farber JL (2002) The course of etoposide-induced apoptosis from damage to DNA and p53 activation to the mitochondrial release of cytochrome c J Biol Chem. 2002 277(19):16547-52. 10.1074/jbc.M110629200 10.1074/jbc.M110629200 11864976

[19] Ding Q, Zhang Z, Liu JJ, Jiang N, Zhang J, Ross TM, Chu XJ, Bartkovitz D, Podlaski F, Janson C, Tovar C, Filipovic ZM, Higgins B, Glenn K, Packman K, Vassilev LT, Graves B (2013) Discovery of RG7388, a potent and selective p53-MDM2 inhibitor in clinical development. J Med Chem 56(14):5979–5983. 10.1021/jm400487c 23808545 10.1021/jm400487c

[20] Carol H, Reynolds CP, Kang MH, Keir ST, Maris JM, Gorlick R, Kolb EA, Billups CA, Geier B, Kurmasheva RT, Houghton PJ, Smith MA, Lock RB (2013) Initial testing of the MDM2 inhibitor RG7112 by the Pediatric Preclinical Testing Program Pediatr Blood Cancer. 60(4):633–641. 10.1002/pbc.24235 10.1002/pbc.24235 PMC3495996 22753001

[21] Ogasawara J, Watanabe-Fukunaga R, Adachi M, Matsuzawa A, Kasugai T, Kitamura Y, Itoh N, Suda T, Nagata S (1993) Lethal effect of the anti-fas antibody in mice. Nature 364(6440):806–809. 10.1038/364806a0 7689176 10.1038/364806a0

[22] Aronin A, Amsili S, Prigozhina TB, Tzdaka K, Shen R, Grinmann L, Szafer F, Edebrink P, Rauvola MA, Shani N, Elhalel MD (2014) Highly efficient, in-vivo Fas-mediated apoptosis of B-cell lymphoma by hexameric CTLA4-FasL. J Hematol Oncol 7:64. 10.1186/s13045-014-0064-6 25227919 10.1186/s13045-014-0064-6 PMC4189725

[23] Makdasi E, Amsili S, Aronin A, Prigozhina TB, Tzdaka K, Gozlan YM, Ben Gigi-Tamir L, Sagiv JY, Shkedy F, Shani N, Tykocinski ML, Dranitzki Elhalel M (2020) Toxicology and pharmacokinetic studies in mice and Nonhuman Primates of the nontoxic, efficient, targeted hexameric FasL: CTLA4-FasL. Mol Cancer Ther 19(2):513–524. 10.1158/1535-7163.MCT-19-0558 31871267 10.1158/1535-7163.MCT-19-0558

[24] Kocik J, Machula M, Wisniewska A, Surmiak E, Holak TA, Skalniak L (2019) Helping the Released Guardian: drug combinations for supporting the Anticancer activity of HDM2 (MDM2) antagonists. Cancers (Basel). 11(7):1014. 10.3390/cancers11071014 10.3390/cancers11071014 PMC6678622 31331108

[25] Tuomela K, Ambrose AR, Davis DM (2022) Escaping death: how Cancer cells and infected cells resist cell-mediated cytotoxicity. 10.3389/fimmu.2022.867098. Front Immunol13:867098 10.3389/fimmu.2022.867098 PMC8984481 35401556

[26] Engels IH, Totzke G, Fischer U, Schulze-Osthoff K, Jänicke RU (2005) Caspase-10 sensitizes breast carcinoma cells to TRAIL-induced but not tumor necrosis factor-induced apoptosis in a caspase-3-dependent manner. Mol Cell Biol 25(7):2808–2818. 10.1128/MCB.25.7.2808-2818.2005 15767684 10.1128/MCB.25.7.2808-2818.2005 PMC1061657

[27] Horn S, Hughes MA, Schilling R, Sticht C, Tenev T, Ploesser M, Meier P, Sprick MR, MacFarlane M, Leverkus M (2017) Caspase-10 negatively regulates caspase-8-Mediated cell death, switching the response to CD95L in Favor of NF-κB activation and cell survival. Cell Rep 19(4):785–797. 10.1016/j.celrep.2017.04.010 28445729 10.1016/j.celrep.2017.04.010 PMC5413585

[28] Hueber AO, Bernard AM, Herincs Z, Couzinet A, He HT (2002) An essential role for membrane rafts in the initiation of Fas/CD95-triggered cell death in mouse thymocytes. EMBO Rep 3(2):190–196. 10.1093/embo-reports/kvf022 11818332 10.1093/embo-reports/kvf022 PMC1083963

[29] Kim JW, Wieckowski E, Taylor DD, Reichert TE, Watkins S, Whiteside TL (2005) Fas ligand-positive membranous vesicles isolated from sera of patients with oral cancer induce apoptosis of activated T lymphocytes. Clin Cancer Res 11(3):1010–1020 15709166

[30] Desbarats J, Newell MK (2000) Fas engagement accelerates liver regeneration after partial hepatectomy. Nat Med 6(8):920–923. 10.1038/78688 10932231 10.1038/78688

[31] Chen L, Park SM, Tumanov AV, Hau A, Sawada K, Feig C, Turner JR, Fu YX, Romero IL, Lengyel E, Peter ME (2010) CD95 promotes tumor growth. Nature 465(7297):492–496. 10.1038/nature09075 20505730 10.1038/nature09075 PMC2879093

[32] Abrahams VM, Straszewski SL, Kamsteeg M, Hanczaruk B, Schwartz PE, Rutherford TJ, Mor G (2003) Epithelial ovarian cancer cells secrete functional Fas ligand. Cancer Res 63(17):5573–5581 14500397

[33] Annibaldi A, Walczak H (2020) Death receptors and their ligands in Inflammatory Disease and Cancer. Cold Spring Harb Perspect Biol 12(9):a036384. 10.1101/cshperspect.a036384 31988141 10.1101/cshperspect.a036384 PMC7461759

[34] Łasut-Szyszka B, Rusin M (2023) The wheel of p53 helps to drive the Immune System. Int J Mol Sci 24(8):7645. 10.3390/ijms24087645 37108808 10.3390/ijms24087645 PMC10143509

