# Supplementary material for: Strong activation of p53 by actinomycin D and nutlin-3a overcomes the resistance of cancer cells to the pro-apoptotic activity of the FAS ligand
Source: Apoptosis. 2024 Jul 28;29(9-10):1515–28. doi: 10.1007/s10495-024-02000-0 (PMC11416401; doi:10.1007/s10495-024-02000-0)
Supplement: Supplementary file 1 — Supplementary Material 1 [file 10495_2024_2000_MOESM1_ESM.docx]

**Supplementary information**

**Supplementary figures**

**
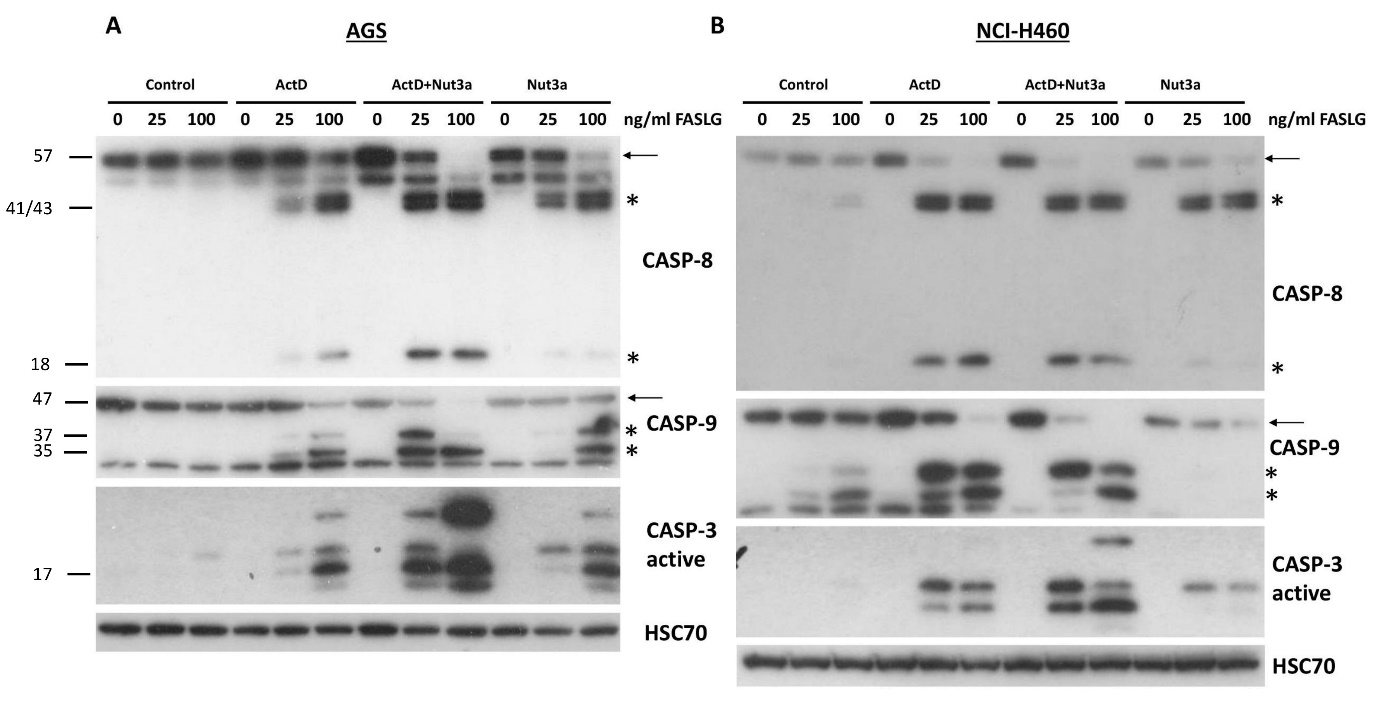
**

**Figure S1. Actinomycin D and nutlin-3a cooperate in sensitizing cells to the pro-apoptotic activity of FASLG in AGS and NCI-H460 cell lines.** AGS (**A**) and NCI-H460 (**B**) cell lines were exposed as indicated (ActD, Nut3a, ActD+Nut3a for 45 hours, FASLG for 2.5 hours) and the expression of caspases was detected by Western blotting.

**
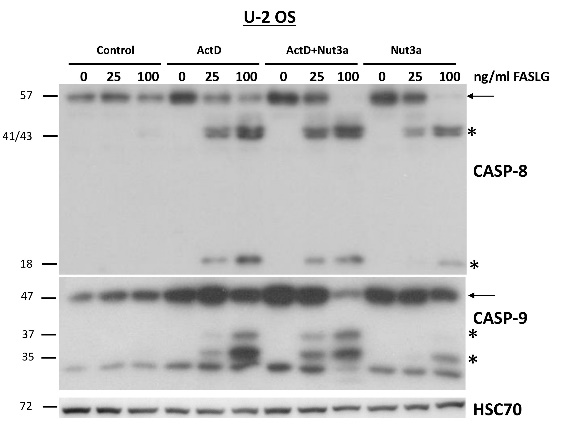
**

**Figure S2.** **Actinomycin D and nutlin-3a cooperate in sensitizing U-2 OS cells to the pro-apoptotic activity of FASLG.** U-2 OS cells were treated as described in the legend of Fig. S1.


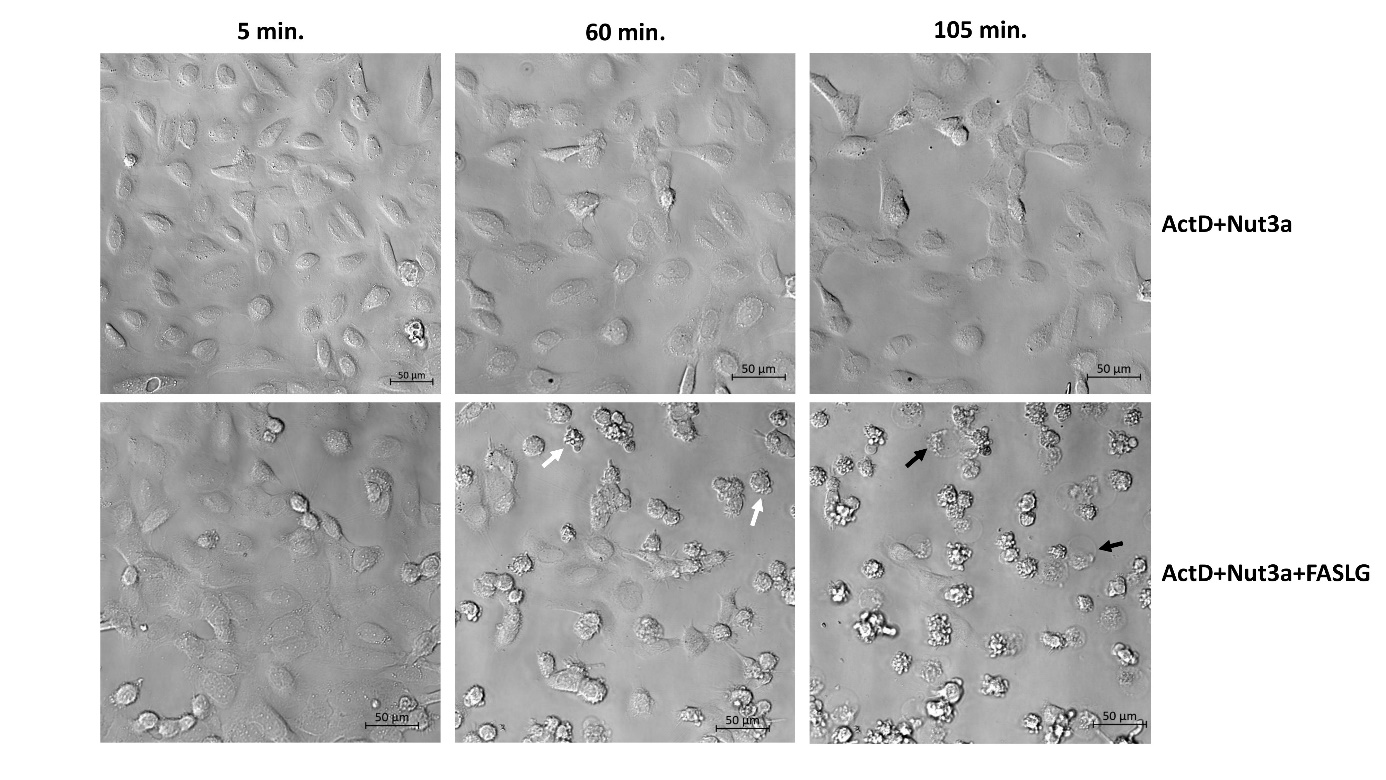
**Figure S3. Microscope observations reveal frequent death of cells pre-exposed to actinomycin D and nutlin-3a and incubated with FASLG.** U-2 OS cells were pre-exposed to ActD+Nut3a for 46 hours and then incubated with or without 100 ng/ml FASLG. At the indicated times, the cells on the same fragment were photographed. White arrows mark examples of cells with early apoptotic morphology, black arrows mark the cells with late apoptotic/necrotic morphology.


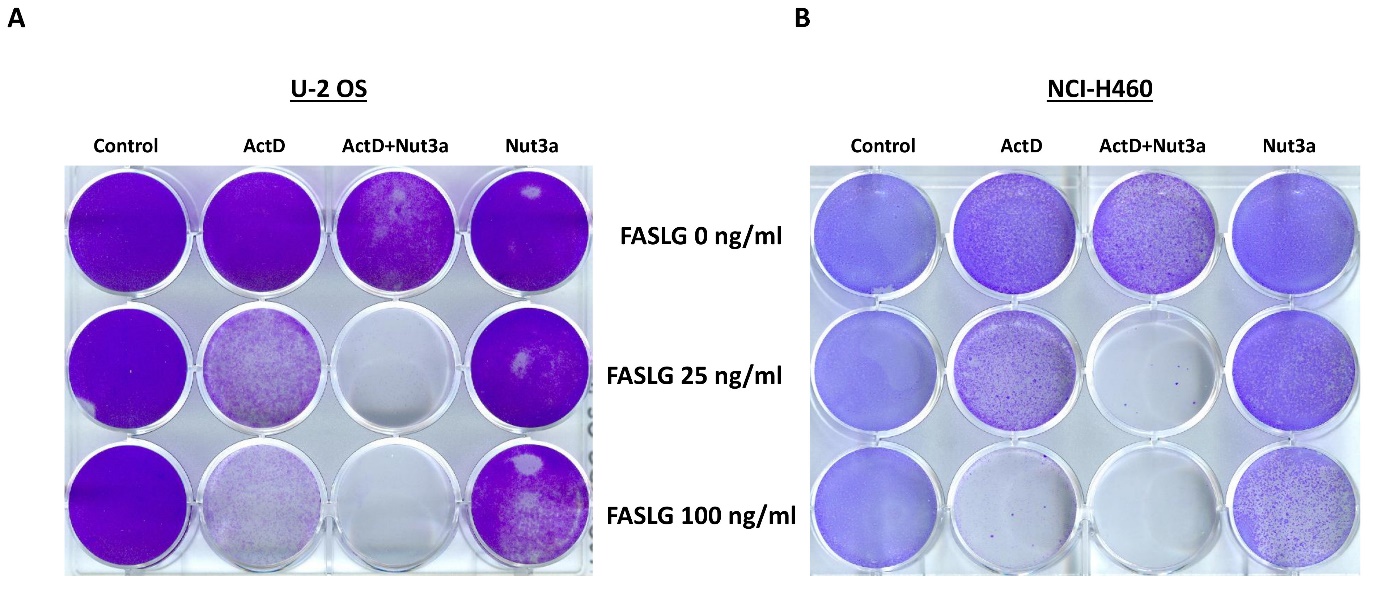


**Figure S4. The cooperation between actinomycin D and nutlin-3a can be visualized by staining of U-2 OS (A) and NCI-H460 cells (B).** The cells attached to the wells of the culture plate (stained by crystal violet) following the indicated treatment regimen. The cells were pretreated with drugs for 46 hours and left untreated (the top row) or exposed to the indicated concentrations of FASLG for 5 hours. Subsequently, surviving cells were allowed to recover for 70 hours before fixation and staining.


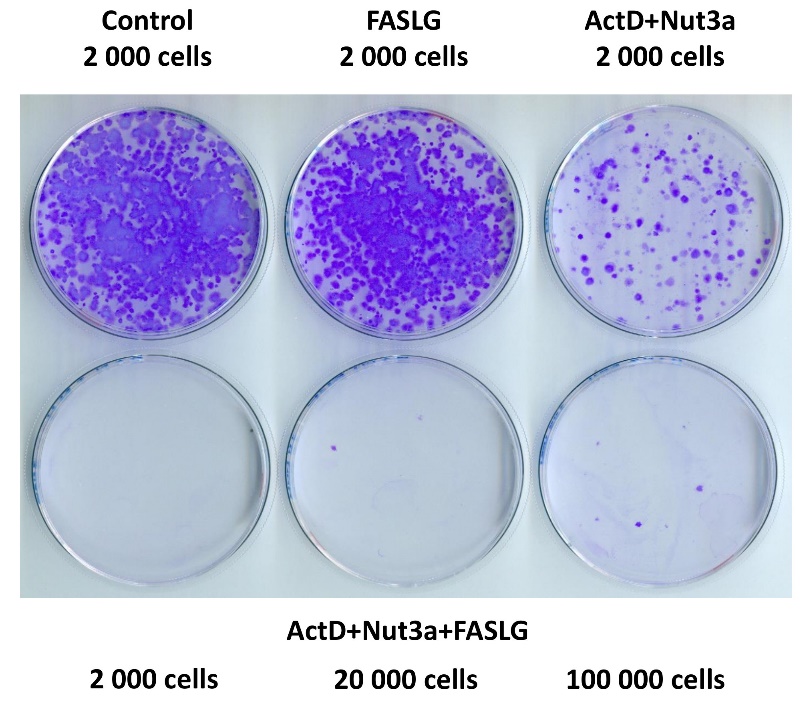


**Figure S5. The clonogenic assay helps to compare the number of cells surviving the treatment with ActD+Nut3a *versus* ActD+Nut3a+FASLG.** A549 cells were exposed to ActD+Nut3a for 45 hours or cultured in control conditions. Subsequently, the cells were trypsinized, counted, and the indicated number of cells were seeded onto a 6 cm culture plate with or without FASLG at 25 ng/ml concentration for 15 hours. Next, the medium was changed, and the cells were allowed to form clones for 14 days. Out of 2000 cells exposed to ActD+Nut3a, 155 clones appeared, whereas in the case of ActD+Nut3a +FASLG treatment, 2000 cells formed 0 clones, 20 000 cells formed 2 clones, and 100 000 cells formed 6 clones. This helps to estimate that, in the case of cells exposed to ActD+Nut3a, the FASLG reduced the clonogenic potential by a factor of approximately 1000. In the case of cells growing in control conditions (DMSO), FASLG did not reduce the clonogenic potential noticeably.

**
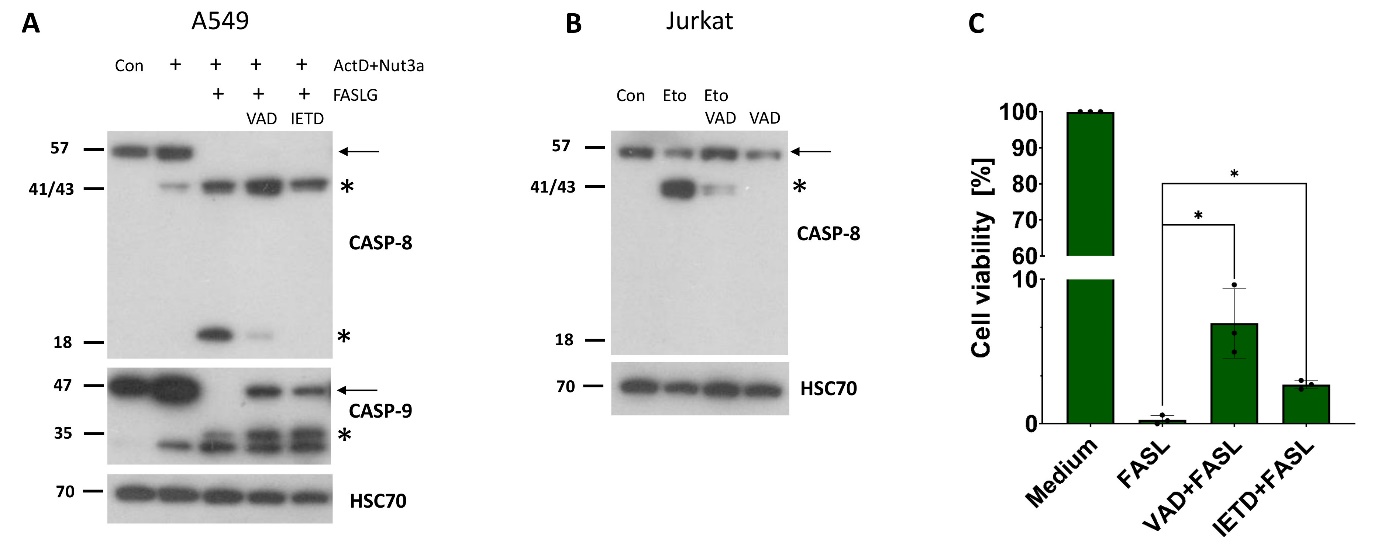
**

**Figure S6. The caspase inhibitors reduce but not block the cell death induced by the treatment with ActD+Nut3a and FASLG. A.** A549 cells were exposed to ActD+Nut3a for 45 hours or were mock-treated (Con). Subsequently, some of the cells were pre-treated for 1 hour with the caspase inhibitors, either Z-VAD-FMK (VAD, pan caspase inhibitor) or Z-IETD-FMK (IETD, caspase-8 inhibitor), both at 100 µM concentration, which was higher than suggested by the manufacturer. The treatment with inhibitors was continued after addition of FASLG (10 ng/ml, 2.5 hours). Note the lack of the active caspase-8 form (18 kDa) and the presence of full-length caspase-9 in cells exposed to the inhibitors. **B.** In order to test the activity of the inhibitor in more commonly used settings, Jurkat cells were pre-exposed with Z-VAD-FMK (100 µM) and subsequently the incubation with the inhibitor was continued in the presence of etoposide (25 µM) for 5 hours as indicated on the figure. **C.** The viability of A549 cells measured by MTS assay. Cells were exposed to Act+Nut3a for 45 hours and subsequently were pre-exposed for 1 hour with the inhibitors as indicated. Next, the FASLG was added to the medium (10 ng/ml) and the incubation was continued for 5 hours. The cells were allowed to recover for 24 hours in fresh medium before the MTS assay was performed. The viability of cells exposed only to ActD+Nut3a (the first column) was set as 100%. The graph shows the results of three biological replicates. The statistical significance was calculated by unpaired *t*-test with Welch’s correction (* p<0.05).

**
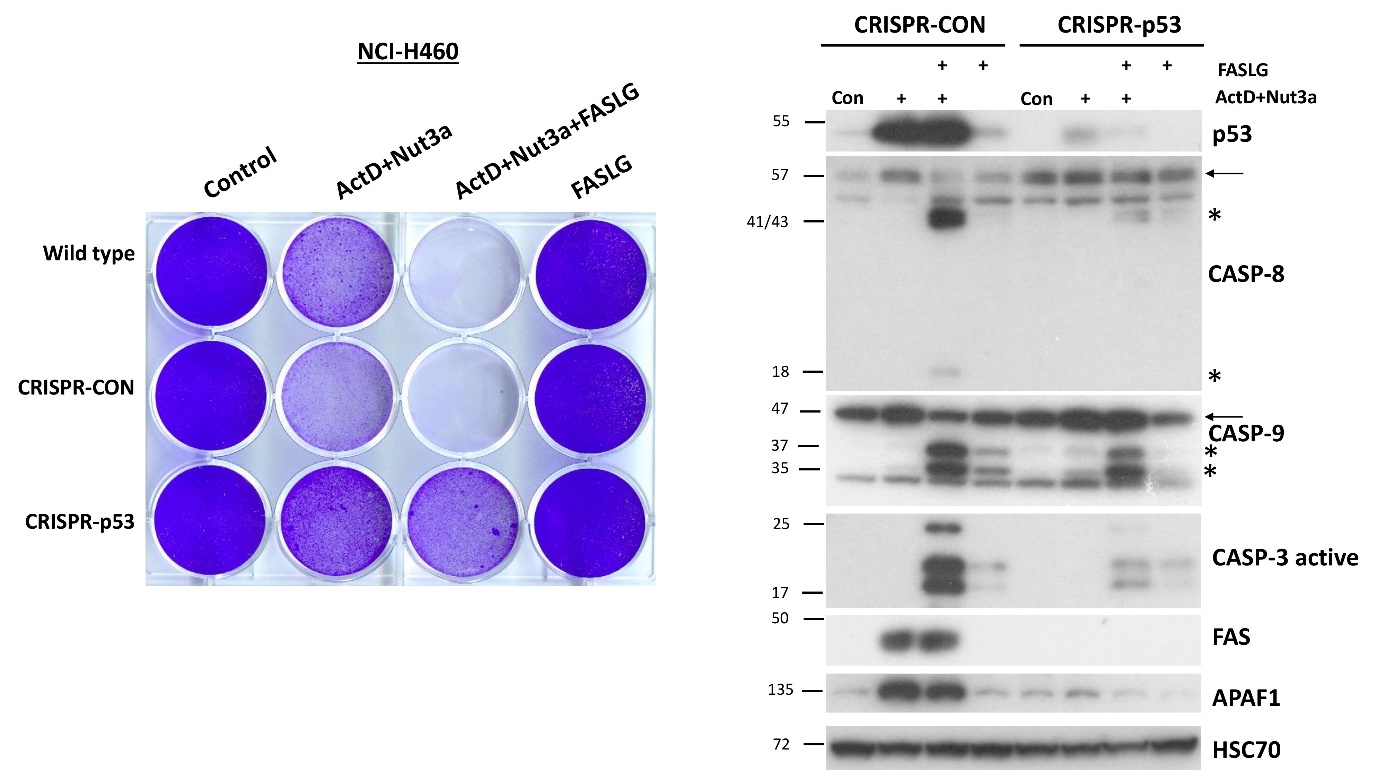
**

**Figure S7. P53 is indispensable for the induction of apoptosis in NCI-H460 cells exposed to ActD+Nut3a+FASLG. A.** The stained cells attached to the wells of the culture plate. The cells of the parental cell line (wild-type), controls for knockdown (CRISPR-CON), and the p53-deficient cells (CRISPR-p53) were pre-exposed as indicated for 45 hours and subsequently treated with FASLG and 100 ng/ml concentration for 5 hours. The surviving cells were allowed to recover for 24 hours. The cells were allowed to overgrow to better visualize the scarcity of cells on the wells exposed to ActD+Nut3a+FASLG. **B.** The expression of the indicated proteins as detected by Western blotting. The p53-proficient and deficient NCI-H460 cells were exposed to ActD+Nut3a for 45 hours followed by exposure to FASLG at 50 ng/ml concentration for 2.5 hours.
